# Impact of fiber-containing enteral nutrition on microbial community dynamics in critically ill trauma patients: a pilot-randomized trial

**DOI:** 10.1186/s12916-025-04511-2

**Published:** 2025-12-29

**Authors:** Mara A. Serbanescu, Mary C. Wright, Mohamed Elebasy, Pixu Shi, Jason W. Arnold, Krista L. Haines, James R. White, Neeraj K. Surana, Paul E. Wischmeyer

**Affiliations:** 1 Department of Anesthesiology, Duke University School of Medicine, DUMC Box 3094 Mail Insert # 11, 2301 Erwin Road, Durham, NC 27710 USA; 2 Department of Biostatistics and Bioinformatics, Duke University School of Medicine, Durham, NC USA; 3 Department of Molecular Genetics and Microbiology, Duke University School of Medicine, Durham, NC USA; 4 Department of Surgery, Duke University School of Medicine, Durham, NC USA; 5 Resphera Biosciences, LLC, Baltimore, USA; 6 Department of Pediatrics, Duke University School of Medicine, Durham, NC USA

**Keywords:** Critical illness, Microbiome, Fiber, Enteral nutrition, ICU, Nutrition, Microbial, Prebiotic, Fructooligosaccharide, ScFOS, Microbiota, Trauma

## Abstract

**Background:**

Gut microbial dysbiosis is common in the intensive care unit and certain derangements, like expansion of *Enterobacteriaceae* and other potential pathogens (pathobionts), are associated with increased morbidity. In other populations, dysbiosis is improved by enteral nutrition supplemented with prebiotic short-chain fructooligosaccharides (scFOS-EN). The impact of scFOS-EN on the microbiota in critical illness is unknown and difficult to predict in a dysbiotic environment. Thus, we conducted a pilot randomized control trial (RCT) in critically ill trauma patients to evaluate the effects of scFOS-EN versus a fiber-free enteral formula (NF-EN) on gut microbial dynamics.

**Methods:**

In this single-center, prospective, double-blind RCT, mechanically ventilated trauma ICU patients received scFOS-EN or a similar fiber-free formula (NF-EN). Microbial communities in longitudinally collected stool samples were characterized using 16S rRNA gene sequencing. We used linear mixed-effects models to assess microbial dynamics in the 10-day study period after scFOS-EN or NF-EN initiation, as well as a time-informed dimensionality reduction method to identify patient-specific temporal responses and clinical correlates and network approaches for microbe:microbe interactions.

**Results:**

A total of 57 stool samples were analyzed from 17 patients (7 NF-EN, 10 scFOS-EN). All participants had profound baseline dysbiosis and received broad-spectrum antibiotics. Compared to NF-EN, scFOS-EN was associated with an accelerated loss of *Bifidobacterium* (− 0.6%/day *p* = .026) and Firmicutes (3.5%/day, *p* < .001) and greater increases in several *Bacteroidaceae* members, with expansion of pathobiont *Enterobacteriaceae* (0.3%/day, *p* = .003) unique to scFOS-EN participants. Detrimental microbial responses to scFOS-EN, including high *Enterobacteriaceae* burden, were dictated by pre-existing and ongoing antibiotic exposure and associated with enhanced microbial competition.

**Conclusions:**

In the dysbiotic gut of critically ill trauma patients, the effect of scFOS-EN is context-dependent. Prior exposure to anaerobic antibiotics appears to modify the microbial response from beneficial to detrimental. These findings challenge a universal approach to prebiotic therapy and underscore the need for personalized nutritional strategies in the ICU.

**Trial registration:**

The trial was prospectively registered at ClinicalTrials.gov (Identifier: NCT03153397; first posted May 15, 2017) prior to participant enrollment and approved by the Duke Health Institutional Review Board (IRB Pro00081414).

**Supplementary Information:**

The online version contains supplementary material available at 10.1186/s12916-025-04511-2.

## Background

The gut microbiota of patients admitted to the intensive care unit (ICU) undergoes rapid and progressive pathologic restructuring, broadly referred to as ICU-associated dysbiosis. This dysbiosis is marked by loss of microbial diversity, depletion of commensal taxa, and expansion of potentially pathogenic bacteria (“pathobionts”) [1]. Expansion of pathobionts, particularly *Enterobacteriaceae*, has repeatedly been associated with an increased risk of nosocomial infection and death in critically ill patients [2–4]. As nosocomial infections affect more than 20% of ICU patients and rank among the leading causes of ICU death [5], identifying interventions that curb pathobiont expansion and mitigate ICU dysbiosis has potential to confer significant clinical benefit. Specifically, therapies that increase abundance of certain beneficial commensals like *Bifidobacterium* (Actinobacteria) and *Ruminococcaceae* and *Lachnospiraceae* (Firmicutes) may be particularly efficacious as these microbes both produce immunomodulatory metabolites like short-chain fatty acids (SCFAs) and compete with pathobionts for nutrients and space [6–8]. Indeed, animal and human studies have shown promise of microbiota-targeted interventions including prebiotic supplementation in mitigating pathobiont expansion and reducing susceptibility to infection [1, 6, 9–11]. However, few clinical studies have explored the impact of these therapies on ICU-associated dysbiosis and their effects in this setting are largely unknown.

Prebiotic fibers like short-chain fructooligosaccharides (scFOS) bypass small-intestinal absorption, thus serving as metabolic substrates for colonic microbes. The effects of prebiotics are context-dependent, and fiber structure, carbohydrate-utilization capabilities of resident microbes, and interactions within the community (e.g., competition and cross-feeding) all determine which microbes benefit from the intervention [12]. ScFOS are particularly attractive prebiotics as their low degrees of polymerization assures they are readily fermented by putatively beneficial *Bifidobacterium* and SCFA-producing Firmicutes [13]. In non-critically ill patients, scFOS and enteral nutrition (EN) supplemented with scFOS (scFOS-EN) is associated with increased abundance of these beneficial commensals, increased SCFA production, and reduced systemic inflammation [9–11]. Nonetheless, in settings of severe dysbiosis or nutrient limitation, readily fermented fiber can be co-opted by *Bacteroidaceae* and pathobiont *Enterobacteriaceae*, microbes that are more resilient to ecologic stress, resulting in their growth at the expense of less resilient community members [14, 15]. ICU patients face ecological pressures such as suboptimal nutrition, antibiotic exposure, and nosocomial infection that alter gut microbiota composition and favor pathobiont expansion [1, 16, 17], thereby potentially influencing which microbes benefit from prebiotic interventions.

Therapeutic enteral nutrition (EN) formulations enriched with scFOS (scFOS-EN) are already used in ICUs worldwide to ameliorate gastrointestinal (GI) intolerance [17–19]. However, the impact of these and other prebiotic interventions on microbial dynamics in the ICU are unknown. No randomized controlled trials (RCTs) have characterized microbiota responses to scFOS-EN in critical illness. Furthermore, while observational studies in ICU patients indicate a positive correlation between intake of dietary fiber and representation of SCFA-producing microbes[20, 21], the only two RCTs that directly assessed impact of supplemental fiber (not scFOS) demonstrated either no effect or a reduction in SCFA-producers [22, 23]. Notably, these studies focused on prebiotic responses of a subset of beneficial commensals rather than the entire community. In the setting of the severely dysbiotic environment of the critically ill, understanding the impact of prebiotic interventions like scFOS-EN on growth patterns and interactions between pathobionts and commensals is crucial.

To address these gaps, we conducted a pilot RCT comparing the effects of scFOS-enriched peptide-based therapeutic EN (scFOS-EN) versus a similar fiber-free formula (NF-EN) on microbial dynamics in ICU patients admitted with severe trauma. We hypothesized that scFOS-EN would differentially impact gut microbial dynamics over a 10-day intervention period, and that its effects may be context-dependent, shaped by baseline microbial disturbances and clinical factors like exposure to antibiotics. Using 16S ribosomal RNA (rRNA) gene sequencing of longitudinal stool and oral samples collected after initiation of study EN, we first investigated the impact of scFOS-EN on diversity and individual microbial dynamics. Then, using advanced time-informed and network-based analyses, we show that gut microbial responses are interconnected, and significantly associated with carbohydrate intake and antibiotic exposure. This pragmatic study provides novel insight into gut microbial responses in the ICU and suggests that under the ecologic pressures sustained by pre-existing (anaerobic and aerobic) antibiotic use, administration of scFOS-EN may favor the expansion of resilient colonizers (*Bacteroides*) and pathobiont *Enterobacteriaceae* at the expense of less resilient, putatively beneficial, commensals.

## Methods

This pilot, single-center, prospective, randomized controlled trial assessed the effects of scFOS administration—delivered as NutraFlora® (P-95, GF2-GF4) in a standard peptide-based therapeutic EN (Vital AF 1.2 Cal)—versus a fiber-free, nutritionally similar, EN formula (Osmolite 1.2 Cal) on gut and oral microbiota in critically ill trauma patients. The trial was registered (ClinicalTrials.gov, NCT03153397) and approved by the Duke Health Institutional Review Board ((IRB) Pro00081414). The primary endpoints were changes in relative abundances of microbial community members following the initiation of scFOS-EN versus NF-EN; additionally, safety and clinical outcomes were also recorded. This study adheres to the Consolidated Standards of Reporting Trials (CONSORT) guidelines (see Additional file 1 for complete CONSORT 2025 checklist).

### Participants

The study was performed at Duke University Hospital in Durham, North Carolina, a level I trauma center in the southeastern USA. Adults 18–80 years old admitted to the neuro or surgical ICU due to major trauma were screened. To be eligible for enrollment, subjects needed to be mechanically ventilated and require enteral nutrition, have an expected length of ICU stay > 3 days, and expected survival > 2 days, as assessed by the treating physician. Exclusion criteria included the following: injury to the GI tract; previous hospitalization or receipt of antibiotic therapy in the 4 weeks prior to the current admission; receipt of probiotics or prebiotic fiber-containing EN within 7 days of enrollment; prior organ transplantation; and chronic conditions with end-stage liver or kidney disease and/or use of chronic immunomodulatory therapies. As a pilot study, we aimed to enroll a cohort size of *n* = 10 per group to evaluate feasibility, which is comparable to other related published studies [22, 23].

### Intervention

After obtaining written informed consent from legally authorized representatives, patients were randomized to receive either Vital® AF 1.2 Cal (1200 cal, 75 g protein, and 111 g carbohydrate, including 5.1 g dietary fiber supplied through NutraFlora® scFOS per liter) (i.e., ScFOS-EN) or Osmolite 1.2 Cal, a fiber-free but otherwise nutritionally similar formula (1200 cal, 55 g protein, 156 g carbohydrate, 0 g dietary fiber per liter) (i.e., NF-EN) (both from Abbott Nutrition, Chicago, IL). Full nutritional information for both EN formulas, including macronutrient profiles and detailed composition of the NutraFlora® scFOS (P-95; consisting of 1-kestose (GF2), nystose (GF3), and fructosyl-nystose (GF4)), is provided in Additional file 2. Upon enrollment, participants were randomized using a computer-generated random allocation in sequentially numbered envelopes. One unblinded clinical research coordinator selected the assigned study EN (NF-EN or scFOS-EN), covered the label and delivered it to the ICU nurse. Other research staff and providers remained blinded to the intervention type apart from the rounding dietician, who determined the formula rate and addition of supplemental protein. Nutrition delivery was tailored to patient-specific targets as per an institutional nutrition assessment protocol considering age, body mass index (BMI), ventilation status, and energy received through medications [18]. As per protocol, no additional dietary fiber was administered during the intervention period, and no other type of nutrition was allowed during study EN administration other than supplemental protein. Participants received study formula for 10 days, or as indicated by the treating physician. Day of study EN initiation was considered Day 0 and the study intervention period was considered to be 10 days unless terminated early due to death or transition to comfort/hospice care. To address safety, clinical outcomes were prospectively monitored by the study team. All adverse events (AEs) were recorded using standardized data collection forms, in accordance with the trial registration. All AEs (such as intermittent fever, tachycardia, new infections, and mortality) were subsequently reviewed by the study team and were determined to be unrelated to the study intervention and consistent with the expected clinical trajectories and complications of a critically ill trauma population.

### Data and sample collection

Data on nutrition intake, including time of EN initiation, hourly rate and volume, and feeding interruption, and supplemental protein was assessed daily by data extraction from electronic nursing flow sheets. The total volume of formula was calculated for each 24 h period (7–7am or 7–7 pm) after randomization to reflect daily intake beginning with Day 0. Volumes of EN administered and formula-specific nutrient profiles were used to calculate daily intake (kilocalories (kcal)); macronutrients (protein—including supplemental protein—carbohydrates, and fats); added sugars; and fiber (derived exclusively from scFOS). Calculations were based on adjusted body weight (kgAdj) for participants with body mass index (BMI) > 30 kg/m2 [19]. Daily averages represent mean values across all 24-h periods during which EN was received (see Additional file 3 for detailed clinical data including participant-specific daily nutrition logs and averages). Research staff also recorded demographic information, admission diagnosis, Acute Physiology, and Chronic Health Evaluation (APACHE) II scores, basic anthropomorphic measurements, and antibiotic administration and microbiologic culture data from time of hospital admission to end of the study period. Antibiotics provided had broad-spectrum activity and were classified into two types of exposures: antibiotics with aerobic-spectrum activity only (primarily vancomycin, cefazolin, bacitracin, cefepime, ceftriaxone) or antibiotics that also were effective against anaerobes, regardless of aerobic coverage (primarily piperacillin-tazobactam, metronidazole) (see Additional file 3).

Sample collection was performed by trained research personnel over the subsequent 10-day study period. Fecal samples were collected opportunistically within 1 h of nurse notification of a stooling event, while anterior tongue swabs for oral samples were performed every other day. Only patients who received one of the study EN formulas for at least 24 h and contributed at least one fecal and one oral sample after formula initiation were included in the final analysis (modified intent-to-treat analysis).

### 16S rRNA amplicon sequencing

Samples were submitted to the Duke Microbiome Center’s Microbiome Core Facility for extraction of deoxyribonucleic acid (DNA) and library preparation. All samples were processed concurrently to avoid batch effects. Briefly, DNA was extracted from fecal and oral samples using the Qiagen PowerSoil Pro DNA Extraction Kit on the Qiagen QIAcube HT automated platform following the manufacturer’s protocols. DNA concentration was quantified with a Qubit fluorometer, and purity was assessed using a NanoDrop spectrophotometer prior to normalization to 1.0 ng/µL for 16S rRNA gene amplicon library preparation. The 16S rRNA gene V4 hypervariable region was amplified using the 515F–806R primer pair containing 5′ Illumina adapter sequences, unique Golay barcodes for multiplexing, and primer pads/linkers per the Earth Microbiome Project (EMP) protocol [24, 25]. Barcoded amplicons were purified with AMPure XP (polymerase chain reaction (PCR)) beads, quantified via Qubit, pooled in equimolar concentrations, and sequenced on an Illumina MiSeq instrument using a 2 × 250 bp paired-end run at the Duke Sequencing and Genomic Technologies Shared Resource. Bioinformatics workflows for data processing and analysis were conducted in R statistical software (v.2023.03.1 + 446, R Foundation for Statistical Computing, Vienna, Austria). Sequencing reads were demultiplexed, quality filtered, denoised, and rarefied to an even level of coverage (25,000 reads) using the DADA2 pipeline[26]. Alpha-diversity was assessed by Shannon index and observed amplicon sequence variant (ASV) counts. Taxonomic classification of unique amplicon sequence variants was performed using Silva-138 classifier with a 99%-identity cutoff [27].

### Statistical analysis

#### Longitudinal analysis of diversity and composition

For primary analyses, multivariable linear mixed effects models with repeated measures (LMMs) were used to evaluate changes in alpha diversity and taxonomic abundance. LMMs included fixed effects for formula type, time (as a continuous variable representing days from study EN initiation), and their interaction, with a random intercept for each participant to account for repeated measures. Sex was included as a covariate in all models to account for baseline imbalance. Linear effects for time were selected given optimal fit by model Akaike Information Criterion (AIC) and residual diagnostics in assessments of Shannon Index using functional form for time of linear, quadradic, or cubic functions. The models demonstrated how microbial values in each group (NF-EN and scFOS-EN) changed over time by estimating slopes (i.e., the beta coefficient for time), which reflect the average within-subject rate-of-change per day. The group-by-time interaction term between scFOS-EN and time further allowed us to identify if the changes over time differed between the formula groups—i.e., whether scFOS-EN was associated with a greater increase or decrease (i.e., accelerated rise of accelerated decline) of that microbial value compared to its trajectory in NF-EN. Fitted models were also used to generate participant-specific predictions including estimated Shannon Index at Day 0 (e.g., “D0 Shannon estimates”) to contextualize initial dysbiosis and endpoint (Day 10) values of *Enterobacteriaceae* (“predicted *Enterobacteriaceae* abundance”) for use in downstream correlation analyses. To identify the most clinically relevant changes, results focus on taxa with mean percent relative abundances (r.a.) ≥ 0.1%; full model outputs for these and less abundant taxa (< 0.01%) are reported in Additional file 4. Additionally, to account for potential imbalance in pre-study admission time between groups, a secondary analysis adding an adjustment term for “days post-ICU admission” was also performed. Significance was set at *p* < 0.05 for multivariable models and all other statistical comparisons. To complement LMMs and for phylum level taxonomy plots, empirical r.a. data from fecal samples was stratified by time interval (0–2 days, 2–5 days, 5–10 days). Day 0–2 was chosen for the first interval to inform the composition of the “baseline microbiota” as scFOS and other prebiotics impact gut microbial composition approximately 36–48 h after initiation [9]. Main figure graphs of LMMs used univariable regression lines derived from LMMs for ease of interpretation, with shading to reflect the 95% confidence intervals (CI), and “raw” r.a. data visualized with locally estimated scatterplot smoothing (LOESS) [28]. LOESS and LMM comparisons and graphs were performed in R.

#### Time-informed dimensionality reduction (TEMPTED)

To overcome the challenges of heterogeneous sampling intervals and identify groups of co-varying microbes sharing common temporal patterns, we used TEMPoral TEnsor Decomposition (TEMPTED), an unsupervised machine learning approach designed for longitudinal data [29]. TEMPTED reduces data complexity by decomposing it into a small number of “principal components” (PCs), each representing a distinct temporal signature driven by changes in co-varying groups of microbes (feature loadings) across participants over time; participant-specific PC scores can then be used to assess relationships between these discrete temporal signatures and clinical features [29]. Unlike methods requiring time-point binning, TEMPTED treats time as a continuous variable, making it uniquely suited to our opportunistic sampling design. TEMPTED was applied to unrarefied, center-log ratio (CLR) transformed ASV counts from participants with longitudinal samples (i.e., at least two samples provided over 24 h apart). Input data was filtered to include ASVs present in at least 20% of samples and at least two time points. The analysis was applied first to the combined cohort (NF-EN + scFOS-EN) to identify overarching dynamics and subsequently to the scFOS-EN group alone to investigate intervention-specific heterogeneity. Participant-specific PC scores were then correlated (Spearman’s rho or Wilcoxon Rank Sum) with a pre-defined set of clinical variables (significance *p* < 0.05) (see Additional file 3 for list of variables assessed).

#### Microbial network analysis

To assess the influence of formula type on community-wide ecologic associations, NF-EN and scFOS-EN gut microbial networks were constructed using MicNet toolbox [30]. Cross-sectional networks were constructed using fecal samples collected within the days 5–10 interval (if multiple available, last sample was used) for each participant to maximize effect of formula type and assure balanced representation. Input ASVs were filtered to include those present at ≥ 0.1% r.a. in at least 20% of samples. We evaluated global network properties, tripartite interaction motifs (competitive [− − +] vs. cooperative [+ + +]), and co-occurrence clusters identified using unsupervised Louvain clustering. Microbe:microbe correlations were estimated using Sparse Correlations for Compositional Data (SparCC), a compositionally-aware algorithm that integrates log transformation and is ideally suited for low diversity data [31]. SparCC correlations were considered significant at an absolute correlation coefficient > 0.4 and a false discovery rate–adjusted *q* < 0.1. Visualizations of network analyses were generated using a locally run version of the MicNet toolbox and GraphPad Prism (v10.1.1; GraphPad Software, San Diego, CA, USA).

## Results

### Patient characteristics and clinical course

**Fig. 1:**
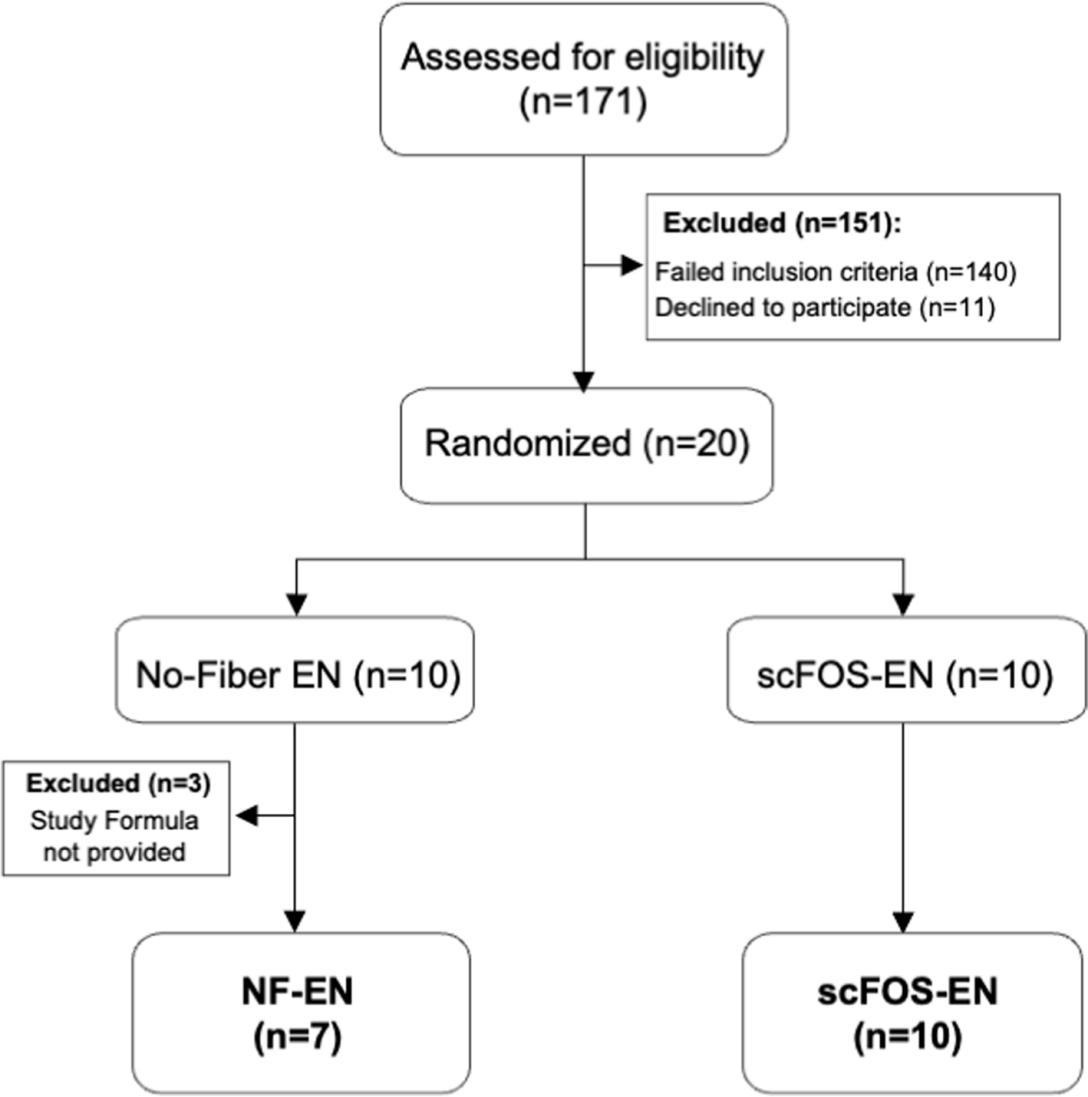
CONSORT flow diagram of patient enrollment. The diagram illustrates the flow of participants through each stage of the randomized controlled trial. A total of 171 critically ill trauma patients were assessed for eligibility, of whom 151 were excluded. Twenty participants were randomized to receive either a no-fiber enteral nutrition formula (NF-EN, *n* = 10) or a formula supplemented with short-chain fructooligosaccharides (scFOS-EN, *n* = 10). Following randomization, three participants were withdrawn from the NF-EN group and samples collected were not analyzed. This resulted in a final cohort of 17 participants (7 NF-EN, 10 scFOS-EN) included in the modified intent-to-treat analysis

Twenty critically ill patients with polytrauma, including traumatic brain injury, were enrolled and randomized between November 2017 and April 2021. Three patients were initially assigned to fiber-free formula group (NF-EN) and withdrawn within 24 h due to rescinded consent (*n* = 1) or protocol deviation by ICU staff (*n* = 2). Thus, a total of 17 patients were included in the final modified intent to treat analysis: 7 NF-EN participants and 10 participants receiving scFOS-supplemented enteral formulation (scFOS-EN) (Fig. 1). From these, 57 fecal and 88 oral samples passed quality control measures and were used for the final analysis. Though fecal sampling was opportunistic and varied by bowel movement timing, similar numbers of samples were obtained in each group (NF-EN: 22; scFOS-EN: 35) (Additional file 5: Fig S1) with all participants submitting at least one fecal sample and all but three contributing longitudinal samples (i.e., $\ge$ 2 samples more than 24 h apart).

Baseline clinical characteristics were generally similar between groups, including APACHE II scores consistent with moderate-to-severe illness severity (Table 1). A higher proportion of males was observed in the scFOS-EN group, a factor that was statistically accounted for as a covariate in all subsequent multivariable models. Initiation of study formula (study EN) occurred a median of 2 days after ICU admission. Caloric delivery was comparable across groups, but below clinical targets and no participant received more than an average of 20 kcal per kg per day (kcal/kg/day) during the study period (Table 1, Additional file 3). As expected, dietary fiber intake differed between groups, with scFOS-EN patients receiving 5.2 g per day (g/day) of fiber versus 0 g/day in NF-EN. Additionally, consistent with differences in formula macronutrients, participants in the scFOS-EN group received fewer total carbohydrates than those in the NF-EN group (Table 1).

**Table 1:**
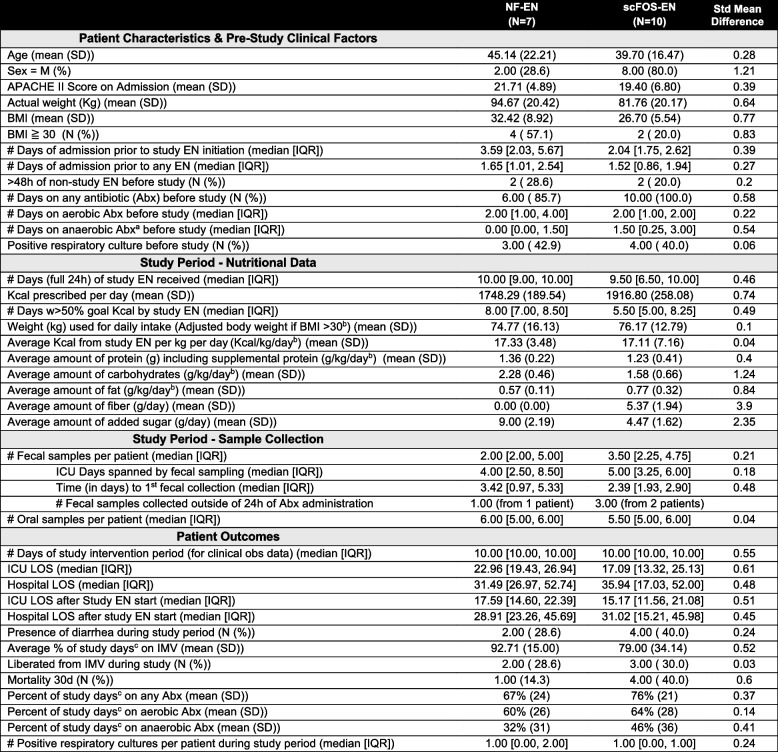
Participant Characteristics

*Acronyms*: *Abx* antibiotics, *EN* enteral nutrition, *IQR* interquartile mean, *LOS* length of stay, *SD* standard deviation

^a^Anaerobic Abx refers to those with anaerobic coverage regardless of aerobic activity (e.g. Piperacillin-Tazobactam & Metronidazole)

^b^Weight for participants with BMI ≧ 30 used Adjusted Body Weight (AdjBW = IBW + 0.4*(Actual Body Weight – Ideal Body Weight)

^c^Percent of study days = (# days of [therapy] ÷ total # of days in study intervention period)*100

**Fig. 2:**
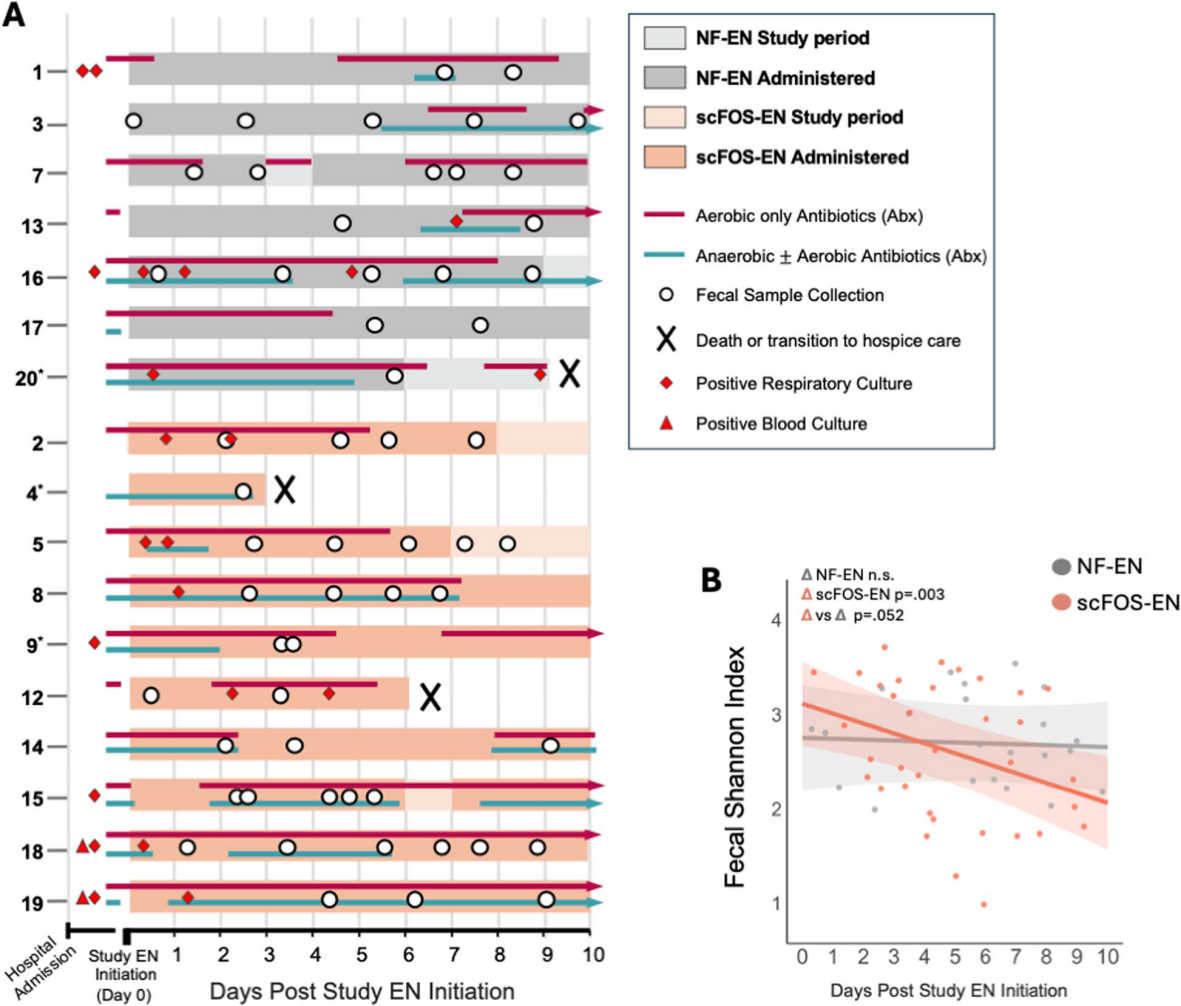
Patient-level clinical timelines and gut microbial alpha diversity dynamics.** A** Swim-lane plot visualizing the clinical course for each of the 17 participants included in the final analysis, aligned by day of study enteral nutrition (EN) initiation (Day 0). Each horizontal lane represents an individual participant, identified by a unique ID. The plot details the timing of EN administration (NF-EN or scFOS-EN), fecal sample collection, administration of antibiotics with aerobic-only versus anaerobic-plus-aerobic coverage, positive blood and respiratory cultures, and denotes timing for those who died or transitioned to hospice care during the intervention period. Events in study intervention period (D0 to Day 10) drawn to scale with each day representing a 24 h period; events prior to D0, not to scale. **B** Trajectories of fecal Shannon diversity over the 10-day study period for the NF-EN (grey) and scFOS-EN (orange) groups (NF-EN *n* = 22 samples, scFOS-EN *n* = 35). Solid lines represent the mean slope of change estimated from linear mixed-effects models. Shaded areas indicate the 95% confidence interval; *p*-values reflect the significance of the within-group trend over time (Δ) and the between-group comparison of slopes (Δ scFOS-EN vs Δ NF-EN) from the group-by-time interaction term

Figure 2A provides a granular view of individual participant timelines, study EN administration, timing of fecal sample collection, antibiotic exposure before and during the study period (aerobic only vs. with anaerobic coverage), and positive blood and respiratory culture data (see Additional file 3 for all participant-level clinical variables). Antibiotic exposure was prevalent with aerobic antibiotics provided to 6 of 7 NF-EN and all 10 scFOS-EN participants before study initiation (pre-study) and to all participants during the study intervention period (Fig. 2A). The average percent of study days spent on antibiotics was also similar between groups, including any antibiotic exposure (67% of total study days in NF-EN vs 76% in scFOS-EN); aerobic-only antibiotics (60% of days in NF-EN vs 64% in scFOS-EN), and anaerobic-active antibiotics (32% of days in NF-EN and 46% in scFOS-EN) (Table 1, Additional file 3). Clinical outcomes were also similar among NF-EN and scFOS-EN participants and indicated high severity of critical illness: only 2 of 7 NF-EN and 3 of 10 scFOS-EN participants were liberated from invasive mechanical ventilation (IMV) during the study period; 3 participants died or transitioned to hospice during the study (1 NF-EN and 2 scFOS-EN); and, among survivors, ICU length of stay (LOS) after study EN start ranged from 9 to 37 days (Table 1, Additional file 3).

### scFOS-EN is associated with declining gut microbial diversity

To assess the impact of scFOS-EN on the gut microbiota, we used multivariable linear mixed-effects models (LMMs) to evaluate within-group changes over time from study EN initiation (Day 0), with group-by-time interaction term to identify the effect attributable to scFOS-EN. In NF-EN participants, microbial diversity remained stable over the study period. In contrast, scFOS-EN participants demonstrated a significant decline in both Shannon diversity (loss of − 0.10/day of study period, 95% CI [− 0.17, − 0.03], *p* = 0.003) and the number of observed ASVs (− 4.2/day, 95% CI [− 6.6, − 1.8], *p* = 0.0005) (Fig. 2B). The group-by-time interaction term approached significance, indicating that scFOS-EN accelerated the loss of diversity by an estimated − 0.092/day beyond the change observed in NF-EN (95% CI [− 0.184, 0.001], *p* = 0.059), suggesting a formula-specific effect.

To contextualize these findings, we used model-based predictions of Day 0 Shannon Index (D0 Shannon estimates) to estimate the baseline microbial state for each participant. D0 Shannon estimates were similar between NF-EN and scFOS-EN participants and ranged from 2.5 to 3.5, consistent with substantial dysbiosis at study entry (Additional file 5: Fig. S2). Correlation analyses revealed that D0 Shannon estimates were significantly associated with several pre-study clinical exposures, with lower Day 0 diversity correlated to longer duration of hospitalization prior to study enrollment (*r* = − 0.671, *p* = 0.004), a greater number of pre-study antibiotic days (*r* = − 0.582, *p* = 0.01), and presence of a positive respiratory culture (*r* = − 0.599, *p* = 0.01)*.*

### scFOS-EN is associated with distinct alterations in gut microbial composition

**Fig. 3:**
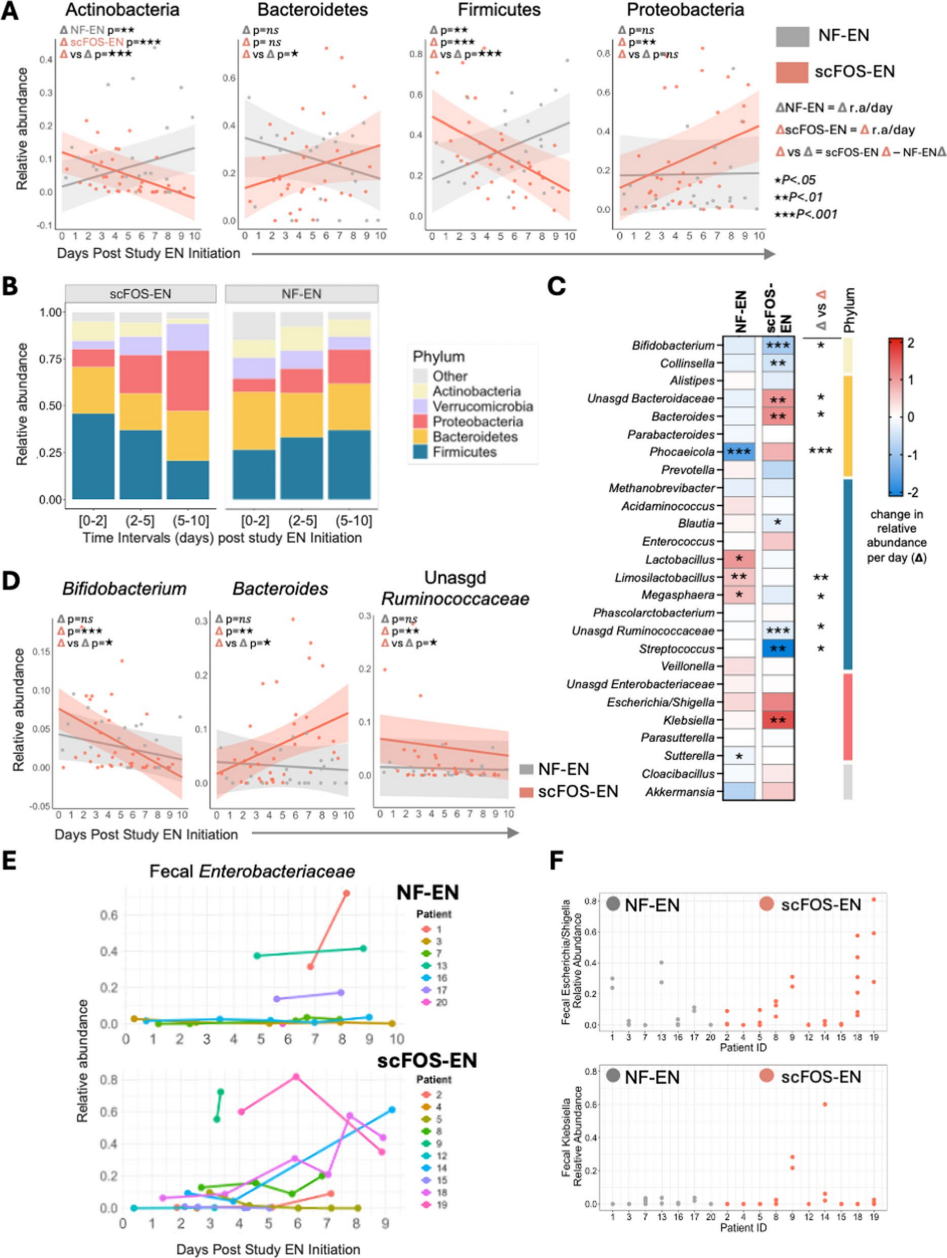
scFOS-EN administration is associated with significant alterations in gut microbial composition.** A** LMM-estimated trajectories of the four dominant phyla over the 10-day study period. Lines represent the mean slope of change in relative abundance (r.a.) for NF-EN and scFOS-EN groups, with shaded areas indicating the 95% CI. *p*-values reflect the significance of the within-group trend (Δ) and the between-group interaction (Δ vs Δ). **B** Mean relative abundance of dominant phyla across three time intervals: baseline (Day 0–2) (*n* = 3 NF-EN samples, *n* = 3 scFOS-EN samples), early intervention (Days 2–5) (*n* = 4 NF-EN, *n* = 18 scFOS-EN) and late intervention (Days 5–10) (*n* = 15 NF-EN, *n* = 15 scFOS-EN). **C** Heatmap of the estimated daily change in relative abundance (%Δ r.a./day) for key genera from LMMs. Columns show the within-group trend for NF-EN, the within-group trend for scFOS-EN, and the between-group interaction effect (Δ vs Δ). Color intensity reflects the magnitude of change (red = increase, blue = decrease). Asterisks denote statistical significance (*p < 0.05, **p < 0.01, ***p < 0.001). **D** LMM-estimated trajectories for key genera *Bifidobacterium*, *Bacteroides*, and *Unassigned Ruminococcaceae*. **E–F** Individual patient trajectories of *Enterobacteriaceae* family r.a., and contributions of *Enterobacteriaceae* members *Escherichia-Shigella* and *Klebsiella*. All LMMs accounted for repeated measures and were adjusted for sex and time since EN initiation, but graphs depict abundances over time without effects of covariables for ease of interpretation

We next investigated changes in compositional features associated with each formula type, again using LMMs as our primary analysis, and then by assessing average relative abundances per group binning samples in three time intervals: Days 0–2 (when prebiotic effect is expected to be minimal, i.e., pre-intervention); Days 2–5 (early intervention); and Days 5–10 (late intervention). LMMs demonstrated that all four dominant phyla—Actinobacteria, Firmicutes, Bacteroidetes and Proteobacteria—changed over the study period on within-group analyses, with group-by-time interaction demonstrating a significant effect of scFOS-EN on enhancing the loss of Actinobacteria (− 2.6%/day compared to rate of change observed in NF-EN [− 3.7%, − 1.5%], *p* < 0.001) and Firmicutes (− 6.3%/day [− 8.8%, − 3.8%], *p* < 0.001), and accelerating expansion of Bacteroidetes (+ 3.7%/day [0.6%, 6.7%], *p* = 0.023) (see Additional file 4 for all analyses of fecal microbiota). Visualization of average r.a. across time intervals highlighted evidence of pre-existing dysbiosis at Days 0–2 (5–10% community occupation by Proteobacteria, > 40% Firmicutes in both groups), and demonstrated the dynamic phylum-level shifts over time statistically identified by LMMs (Fig. 3B, Additional file 4).

Genus-level analyses further identified changes in numerous taxa over time in both NF-EN and scFOS-EN groups (Fig. 3C, Additional file 4, Additional file 5: Fig. S3), though baseline differences were only estimated to be significant for *Phocaeicola* (lower in scFOS-EN) and *unassigned (U) Ruminococcaceae*. Among notable findings within scFOS-EN participants included an increase in pathobiont *Klebsiella* (+ 1.7%/day [0.5%, 2.8%], *p* = 0.004), and a non-significant upward trend in *Escherichia-Shigella* (both *Enterobacteriaceae* family) (Additional file 5: Fig. S4). Comparisons to rates of change observed in NF-EN by group-by-time interaction demonstrated that scFOS-EN was associated with more rapid declines in *Bifidobacterium* (− 0.6%/day beyond that observed in NF-EN [− 1.1%, − 0.1%], *p* = 0.026); *U-Ruminococcaceae* (− 0.3%/day [− 0.5%, 0%], *p* = 0.023); *Limosilactobacillus* (− 0.6%/day [− 1%, − 0.2%], *p* = 0.003); *Streptococcus* (− 2.1%/day [− 4%, − 0.2%], *p* = 0.035); and *Megasphaera* (− 1%/day [− 1.8%, − 0.2%], *p* = 0.025). Conversely, scFOS-EN accelerated the expansion of *Bacteroides* (+ 1.3%/day [0.2%, 2.4%], *p* = 0.027); *Phocaeicola* (+ 2.4%/day [1.3%, 3.5%], *p* < 0.001); and *U-Bacteroidaceae* (+ 1.3%/day [0.2%, 2.4%], *p* = 0.024), with a trend toward significant interaction effect on *Klebsiella* (+ 1.6%/day [0, 3.1%], *p* = 0.052) (Fig. 3D, Additional file 4). All results persisted in sensitivity analyses adjusting for time between ICU admission and study EN initiation.

Finally, we modeled changes in the *Enterobacteriaceae* family (Proteobacteria phylum) explicitly as high *Enterobacteriaceae* burden has been independently associated with worse outcomes in other ICU studies[4, 32], and numerous members of this family have been shown to be capable of co-opting rapidly digestible fibers [33, 34]. Consistent with genus-level trends, scFOS-EN demonstrated a significant expansion of *Enterobacteriaceae* over the study period (+ 3.0%/day [1.0%, 5.0%], *p* = 0.003), with the group-by-time interaction indicating that relative to NF-EN, an estimated + 2.5%/day increase in *Enterobacteriaceae* was attributable to scFOS-EN (95% CI [− 0.2%, 5.2%], *p* = 0.081). *Enterobacteriaceae* expansion was nearly universal in longitudinal samples and high *Enterobacteriaceae* burden (which we defined as ≥ 20% relative abundance) was present in 29% NF-EN and 50% scFOS-EN participants (Fig. 3E), largely due accounted for by abundances of *Escherichia-Shigella* and *Klebsiella* (Fig. 3F). Furthermore, LMM-based predictions of *Enterobacteriaceae* abundance at Day 10 forecasted that high *Enterobacteriaceae* burden would be present in 90% of scFOS-EN participants by the end of the study period, versus only 29% in NF-EN (*p* = 0.04) (Additional file 5: Fig. S4). Together, these findings indicated not only that scFOS-EN is associated with deleterious compositional changes amongst commensals—favoring expansion of *Bacteroidaceae* members over *Bifidobacterium* and Firmicutes—but also that its effects may be tied to a greater expansion of pathobiont *Enterobacteriaceae*.

#### scFOS-EN has little impact on oral microbial composition

We further evaluated effects of NF-EN and scFOS-EN on oral microbiota, which served as a type of negative control as delivery of study EN inherently bypassed the oropharynx. LMMs of oral samples from NF-EN and scFOS-EN showed minimal changes: the only significantly different compositional change over time was an accelerated rise in *Carnobacteriaceae*-U (a subset of common oral microbes) in the scFOS-EN group (+ 0.3%/day difference [0, 0.6%], *p* = 0.03) (Additional file 5: Fig. S5), consistent with a localized effect of NF-EN and scFOS-EN on gut microbiota composition.

#### Linking interconnected time-dependent gut microbial responses in NF-EN and scFOS-EN to clinical features using TEMPTED

**Fig. 4:**
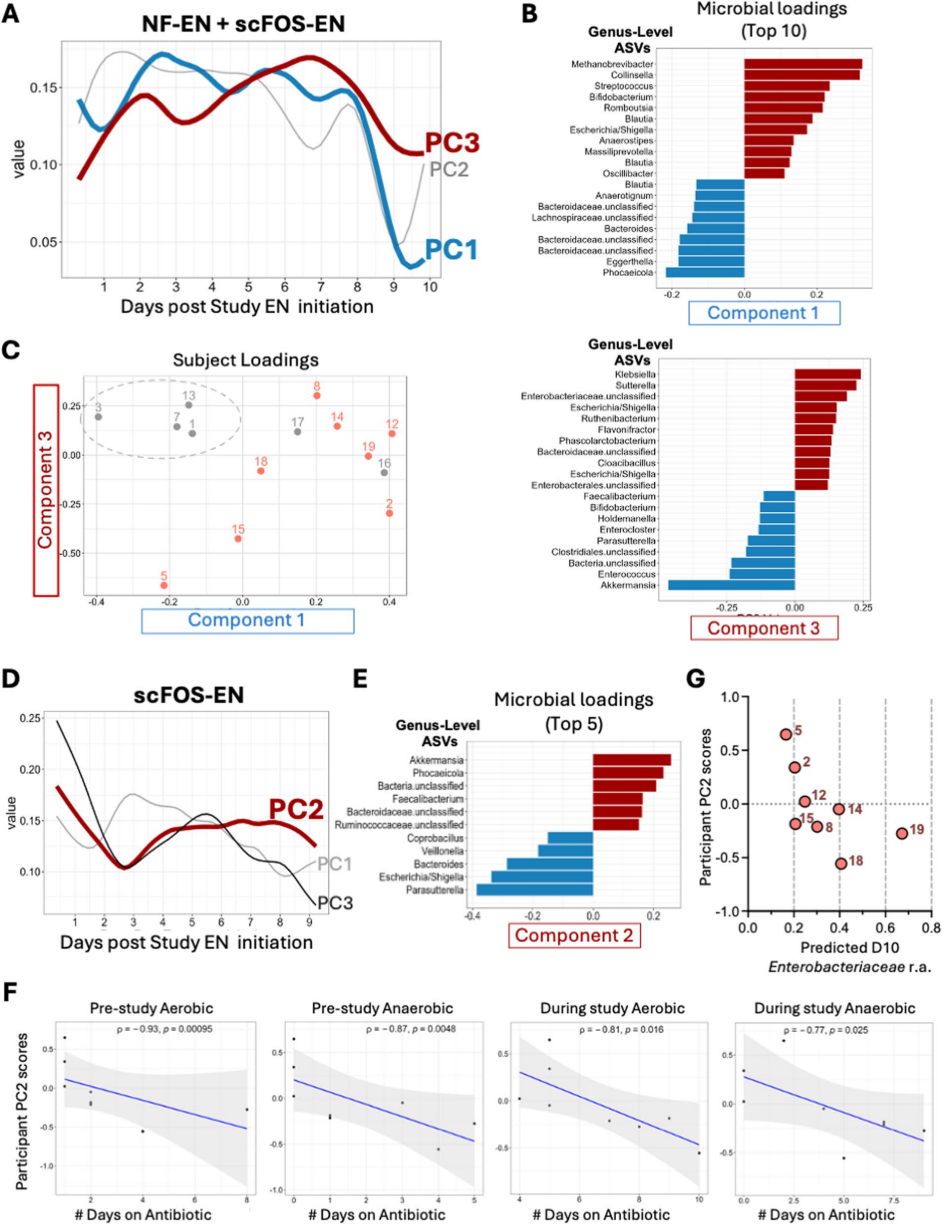
Time-informed dimensionality reduction reveals interconnected microbial signatures and a dichotomous response to scFOS-EN. Results from TEMPoral TEnsor Decomposition (TEMPTED), an unsupervised machine learning method applied to longitudinal ASV-level data. **A**–**C** Results from the combined analysis of all participants. **A** Temporal loadings (i.e., patterns of change over time) for the top three principal components (PCs). **B** Top 10 microbial ASVs with the highest positive (red) and negative (blue) feature loadings for PC1 (associated with infection/inflammation) and PC3 (carbohydrate-driven pathobiont expansion signature). **C** Scatter plot of individual participant scores for PC1 versus PC3. Points are colored by study group (NF-EN: grey, scFOS-EN: orange), NF-EN participants who did not receive pre-study anaerobic antibiotics are highlighted with grey circle. **D**–**G** Results from TEMPTED analysis of only the scFOS-EN group. **D** Temporal loading for PC2, which separates participants into a beneficial (positive) versus detrimental (negative) trajectory. **E** Top 5 microbial ASVs with the highest positive and negative feature loadings for PC2. **F** Spearman correlations between participant PC2 scores and antibiotic exposure, with correlation coefficient (*ρ*) and *p*-value shown for each. **G** Spearman correlations between individual participant PC2 scores and LMM-estimated Day 10 *Enterobacteriaceae* abundance, further demonstrating separation between participants not exposed to anaerobic antibiotics (PC2 > 0) and those exposed (PC2 < 0)

We next sought to determine if the dynamic changes in individual gut community members after NF-EN and scFOS-EN by LMMs reflected interconnected shifts between commensals and pathobionts, particularly in the context of other environmental pressures from antibiotics and changing clinical course. To identify co-varying microbial features over time and their relationships to clinical exposures and enteral formula type, we used TEMPTED, a dimensionality reduction technique specifically designed to accommodate unevenly sampled longitudinal microbiome data [29]. We first applied TEMPTED to ASV-level data from all participants with longitudinal samples (53 samples total from 6 NF-EN and 8 scFOS-EN participants) (Fig. 2A). The top three principal components (PCs) identified by this analysis showed distinct temporal patterns (Fig. 4A) with PC1 and PC3 demonstrating clear clinical relevance.

The first principal component (PC1) was characterized by an early peak followed by slight variation until sharply declining around days 7–8 (Fig. 4A). This component also featured ASVs from genera impacted by scFOS-EN, with positive loading features driving its trajectory that included *Methanobrevibacter* (*ASV31*), *Collinsella* (*ASV37*), *Streptococcus* (*ASV14*), and *Bifidobacterium* (*ASV39*), while taxa with inverse temporal trends (a rapid increase at days 7–8) included *Phocaeicola* (*ASV7*), *Bacteroides* (*ASV60*), and several *U-Bacteroidaceae* (*ASVs 172*, *176*, *264*) (Fig. 4B top, Additional file 4). Participant scores for PC1 were positively correlated with the number of culture events (respiratory/blood) (*r* = 0.60, *p* = 0.026) and were higher in patients with positive respiratory cultures during the study period (mean of 0.234 vs − 0.076 respectively, *p* = 0.03), linking this signature to unresolved infection and pneumonia. In contrast to PC1, principal component (PC3) captured a trajectory of bimodal expansion around day 2 and day 6 of the study period (Fig. 4A). This trajectory reflected dynamics of several *Enterobacteriaceae* and other pathobionts, with top positive loading features including ASVs mapping to *Klebsiella* (ASV4), *Sutterella* (ASV69), and *Escherichia-Shigella* (ASV2, ASV108). Meanwhile, top negative loading features with diametrically opposing temporal trends included *Akkermansia* (*ASV6*), *Enterococcus* (ASV24) as well as beneficial SCFA-producers including *Bifidobacterium* (ASV39) and *Faecalibacterium* (ASV147) (Fig. 4B bottom, Additional file 4). Correlations to clinical variables demonstrated that participant scores for PC3 correlated strongly with carbohydrate intake (*r* = 0.701, *p* = 0.007) and average daily caloric delivery (*r* = 0.789, *p* = 0.001), but not with estimated Day 10 *Enterobacteriaceae* abundance, nor did PC3 scores differ between those with/without high *Enterobacteriaceae* burden. Together, these data suggest that PC3 represents a carbohydrate-driven pathobiont expansion signature, shaped in part, but not exclusively, by *Enterobacteriaceae* members.

While no single PC score differed significantly between the NF-EN and scFOS-EN groups, visualization of participant scores showed that PC1 and PC3 jointly revealed a distinct clustering pattern (Fig. 4C). Most scFOS-EN participants, including those that did not receive pre-study anaerobic antibiotics, are in the top right quadrant of the PC1/PC3 space, suggesting increased representation of microbial patterns associated with both infection and carbohydrate-driven pathobiont expansion. In contrast, a subgroup of NF-EN participants, uniquely characterized by their lack of pre-study anaerobic antibiotic exposure, formed a separate cluster with high PC3 but low PC1 scores, suggesting pathobiont expansion without the corresponding infectious signature (Fig. 4C). Together, these findings provide biological context for compositional changes previously identified by LMMs and underscore key clinical determinants shaping these responses including infection, carbohydrate intake, and potentially, antibiotic exposure.

### Context-specific microbial dynamics in the scFOS-EN group using TEMPTED

To better understand the heterogeneity of microbial responses *within* the intervention arm and identify if these may be shaped by clinical factors like exposure to antibiotics, we next applied TEMPTED exclusively to the longitudinal samples from the scFOS-EN group (32 samples from *n* = 8 scFOS-EN participants). This analysis also identified three principal components driven by microbial loadings distinct from those in the combined group analysis (Fig. 4D–E, Additional file 4). While participant scores for PC1 and PC3 were not tied to clinical features, PC2 scores were exclusively positive among participants without pre-study anaerobic antibiotic exposure, suggesting a divergent response to the scFOS-EN (Additional file 4). PC2 was defined by the rise of commensals such as *Akkermansia* (ASV 6), *Phocaicola* (ASV 7), *Faecalibacterium* (ASV 147), and U-*Ruminococcaceae* (ASV 125), indicating expansion of putatively beneficial commensals; in stark contrast, the opposing detrimental trajectory was defined by the expansion of pathobiont *Escherichia-Shigella* (ASV2) and *Bacteroides* (ASV49) (Fig. 4E, Additional file 4). Correlations with clinical data underscored the importance of antibiotic exposure on these opposing trajectories: positive PC2 participant scores—reflecting greater representation of beneficial microbial features—were *inversely* associated with days of pre-study anaerobic (*r* = − 0.872, *p* = 0.01) and aerobic (*r* = − 926, *p* = 0.004) antibiotic exposure, as well as days of anaerobic (*r* = − 0.711, *p* = 0.033) and aerobic (*r* = − 0.805, *p* = 0.025) antibiotics received during the study period (Fig. 4F), providing strong evidence that the microbial response to scFOS-EN may be fundamentally modified by the pre-existing, antibiotic-induced state of the gut ecosystem. Additionally, participant scores for PC2 (but no other components tested in the combined or scFOS-only analysis) were associated with beneficial clinical outcomes, demonstrating an inverse relationship to percent of days on IMV during the study period (*r* = − 0.764, *p* = 0.036) and total ICU LOS (*r* = − 0.964, *p* = 0.003). Participant PC2 scores were also negatively correlated with predicted *Enterobacteriaceae* abundance at day 10 (*r* = − 0.857, *p* = 0.01) (Fig. 4G) and trended negatively with high *Enterobacteriaceae* burden (*r* = − 0.764, *p* = 0.057). Together this suggests that increases over time of negative PC2 microbial features (i.e., *Escherichia-Shigella*, *Bacteroides*) underlie *Enterobacteriaceae* expansion and may be linked to prolonged ICU stays. Thus, while in a less-disturbed gut, scFOS-EN may support beneficial commensals, in a gut already damaged by broad-spectrum anaerobic antibiotics, the same formula appears to favor the expansion of a detrimental, pathobiont-associated microbial profile.

### Network analysis highlights increased microbial competition in scFOS-EN

Finally, we aimed to identify if the temporal patterns in co-varying microbial features identified in TEMPTED were in fact reflective of a shift in how co-occurring microbes were interacting with each other in response to nutrients introduced by scFOS-EN and NF-EN. Thus, we used Micnet Toolbox to construct co-occurrence networks of NF-EN and scFOS-EN using ASV-level data from the days 5–10 interval fecal samples collected from each participant, providing a cross-sectional view in time of interactions at the peak effect of each intervention [30]. Samples used for NF-EN and scFOS-EN networks were collected at similar times after study EN initiation and demonstrated similar *Enterobacteriaceae* relative abundance between groups, assuring minimal effect of sampling bias (Additional file 5: Fig. S6) While both networks displayed similar overall complexity and density, their underlying ecological structures differed profoundly. The NF-EN network showed that tripartite interactions were balanced between competitive (− − +) (50%) and cooperative (+ + +) (50%). In contrast, in the scFOS-EN network, competitive interactions (− − +) accounted for nearly 75% of all connections, suggesting a far more competitive landscape (Additional file 5: Fig. S7). In NF-EN and scFOS-EN, microbial features of the carbohydrate-responsive, *pathobiont*-associated PC (PC3) clustered together (i.e., *Escherichia-Shigella* (*ASV2*) and *U-Enterobacteriaceae (*ASV 120)), indicating similar patterns of connectivity and nutrient utilization [30]. Nonetheless, only in the scFOS-EN network did this cluster also include *Klebsiella* (ASV 4) and *Sutterella* (ASV 69), potentially due to increased co-occurrence of these features in scFOS-EN (Additional file 5: Fig. S8). Correlation analyses further revealed that while *Enterobacteriaceae* in both networks demonstrated evidence of competition with commensals, these negative interactions were far more prevalent in the scFOS-EN network, with *Escherichia-Shigella* (ASV 2) in particular exhibiting significant negative correlations with a broad range of taxa including Actinobacteria and Firmicutes depleted over time in our LMM and TEMPTED analyses, as well as several *Bacteroidaceae* members (Additional file 5: Fig. S8). Together these data provide a plausible ecological mechanism—direct competition for resources—for the observed decline of these beneficial microbes in the presence of scFOS-EN.

## Discussion

In this pilot RCT of critically ill trauma patients, we found that enteral nutrition supplemented with prebiotic scFOS did not confer the expected benefits observed in healthier populations [10, 11, 27]. Rather than supporting beneficial taxa, it was associated with accelerated loss of *Bifidobacterium* and Firmicutes, expansion of *Bacteroides* and other *Bacteroidaceae* members, and potentially contributed to the expansion of pathobiont *Enterobacteriaceae*. Crucially, our novel temporal analyses reveal that expansion of pathobionts at the expense of beneficial commensals is shaped by carbohydrate intake, ongoing infection, and antibiotics, and that deleterious microbiota changes in scFOS-EN (including high *Enterobacteriaceae* burden) significantly associated with exposure to antibiotics both before and during the intervention period. Together, these findings provide strong evidence that the baseline microbial context dictates the outcome of prebiotic therapy in the ICU.

We and others have previously used culture-independent sequencing techniques to show that gut microbial dysbiosis in the ICU is profound and progressive, with characteristic alterations that include loss of SCFA-producing Firmicutes and expansion of potential pathogens [3, 4, 31, 33, 35]. Here, we demonstrate the crucial role of enteral nutrition in shaping these changes, using a holistic approach to investigate microbial dynamics and interactions following initiation of two widely available EN formulations. Notably, the environmental pressures common among our participants are not atypical; approximately 70% of ICU patients receive antibiotics on any given day [5], and underfeeding is prevalent particularly within the first week of ICU admission [17]. Though ours is the first RCT to our knowledge to evaluate scFOS-EN in critical illness, the deleterious changes we observed echo those from other studies of prebiotics in dysbiotic hosts. In a 22-patient ICU RCT, oligofructose/inulin supplementation led to a decline in *Faecalibacterium*, a key SCFA-producing Firmicutes member [21]. Similarly, in an animal model of antibiotic-induced dysbiosis, FOS supplementation hindered recovery and led to expansion of *Bacteroides* and pathobionts including *Klebsiella* and *Escherichia-Shigella* [34].

To further explore longitudinal patterns, we applied TEMPTED, a novel time-aware dimensionality reduction method [29]. In a combined analysis of both groups, TEMPTED identified a temporal trajectory (PC3) driven by the co-expansion of pathobionts including *Klebsiella*, *Sutterella*, and *Escherichia*-*Shigella*, which was opposed by the beneficial commensals *Akkermansia*, *Bifidobacterium*, and *Faecalibacterium* [6]. This pathobiont-associated signature was highly prevalent across most patients and correlated significantly with carbohydrate delivery, but not fiber intake. This aligns with studies demonstrating that in the setting of dysbiosis and/or carbohydrate restriction, simple carbohydrate availability can fuel opportunistic taxa in the absence of robust fermenters [7, 14]. Notably, pathobionts differ in their carbohydrate preferences, and both *Klebsiella* and *Sutterella* have been shown to utilize complex carbohydrates and longer chain prebiotic fibers, while *E. Coli* favors simple sugars [8, 36, 37]. These preferences may explain co-localization of *Klebsiella* and *Sutterella* as top features of PC3, and the greater co-occurrence of these ASVs in scFOS-EN networks (but not NF-EN) may have contributed to the increase in *Klebsiella* identified in scFOS-EN by LMMs, despite it being responsive to carbohydrates in both formula types.

While PC3 participant scores highlighted carbohydrate-responsive pathobionts, the temporal trajectory associated with *Enterobacteriaceae* expansion was identified in our TEMPTED analysis of scFOS-EN participants alone. Here, we found that high *Enterobacteriaceae* burden was associated with a distinct microbial pattern characterized by greater representation of *Escherichia-Shigella*, and specific *Bacteroides* and *Parasutterella* ASVs (i.e., “negative PC2” loading features) relative to beneficial taxa including *Akkermansia*, *Phocaeicola*, and *Faecalibacterium* (“positive PC2” features). Perhaps more importantly, we identified a striking bifurcation in temporal PC2 microbial responses based on antibiotic exposure. Specifically, participants exposed to pre-study anaerobic agents had a greater representation of “PC2 negative” microbial features, with duration of aerobic and anaerobic antibiotics both before scFOS-EN initiation and during administration also identified as potential modifiers. This may explain why, despite adequate scFOS doses, we failed to see the expected expansion of *Bifidobacterium* and SCFA-producing Firmicutes. In an ecosystem stripped of these key commensals, more resilient taxa may fill this ecologic niche. Indeed, in nutrient-limited environments, certain *E. Coli* and *Bacteroides* species preferentially utilize rapidly digestible carbon sources, leveraging their own growth over other commensals and later, may even compete against each other [15, 38]. This ecological phenomenon is supported by our network analysis, which demonstrated increased competitive interactions in the scFOS-EN group, particularly between pathobiont *Enterobacteriaceae* and diverse commensals, including *Bacteroides*. Collectively, these data suggest that ICU-associated dysbiosis is akin to a “scorched lawn”—damaged by both limited nutrition and broad-spectrum antibiotics. In this barren landscape, adding a prebiotic like scFOS may be like fertilizing weeds: resilient, fast-growing taxa dominate and suppress any remaining commensals, and may even eventually competing against each other for space.

Although our study was not powered for clinical outcomes, the “negative PC2” phenotype was also associated with prolonged ICU stay and duration of mechanical ventilation. This mirrors known relationships between high *Enterobacteriaceae* burden, antibiotic exposure [39–41], and worse clinical outcomes in other ICU studies [4, 32, 42]. Our study adds to this literature by raising the possibility that scFOS-EN, when administered after or during broad-spectrum antibiotic therapy, may enhance microbial features associated with high *Enterobacteriaceae* burden. We further highlight a link between the *Enterobacteriaceae* phenotype in the ICU and the temporal expansion of *Escherichia-Shigella* and resilient colonizer *Bacteroides*, with synergistic expansion of these microbes demonstrated in undernourished children and specifically linked to intestinal inflammation [14].

Notably, the beneficial “PC2 positive” commensals were more abundant in participants with limited antibiotic exposure, and LMMs showed that scFOS-EN accelerated the expansion of *Phocaeicola*, a leading beneficial feature. Together, this suggests a potential beneficial effect of scFOS-EN if applied to more intact gut microbial communities. As antibiotic exposure is difficult to avoid in the ICU, these findings also highlight the potential promise of therapies that reconstitute the gut microbiota, such as synbiotics. In support, a large RCT found that *Lactobacillus* plus FOS reduced neonatal sepsis risk [8], highlighting the importance of co-administration strategies in compromised microbial ecosystems.

Our study has several limitations. First, as a pilot investigation, the sample size is limited, which prevents us from associating microbial responses with clinical endpoints and means our results should be validated in a larger trial. While residual confounding cannot be excluded, the blinded, randomized design and the convergence of findings across LMMs, network analysis, and TEMPTED all strengthen confidence in the key biological signals. Second, we must explicitly acknowledge the methodological challenge of disentangling the specific contribution of scFOS from the potent effects of concurrent antibiotic exposure. Antibiotic administration is a critical and non-randomized co-intervention in the ICU and creates a powerful ecological pressure that, particularly within a pilot-scale cohort, limits the statistical power to fully separate these two effects. While our TEMPTED analysis began to identify divergent microbial responses based on antibiotic use, we cannot definitively de-confound the impact of scFOS from this antibiotic-driven background. Therefore, the complex interplay between antibiotics and the scFOS intervention should be viewed as a key finding that warrants further investigation in larger, adequately-powered studies. Third, we acknowledge the inherent dissimilarities in the EN formulations beyond scFOS. While protein intake was similar, NF-EN participants received more added sugar. Nonetheless, the fact that many changes attributable to scFOS-EN were also key features in our TEMPTED analysis of *only* the scFOS-EN participants underscores the likelihood that these signals were attached to scFOS itself. Finally, we recognize the known limitations of 16S rRNA sequencing, including the inability to quantify total microbial load, assess mucosa-associated communities, and identify functional profiles. Further investigations using qPCR, metagenomic sequencing, and transcriptomics should be implemented in future studies with larger sample sizes.

## Conclusion

In conclusion, this study underscores that the effects of prebiotic fiber—and carbohydrates in general—in enteral nutrition are not universal, but context- and community-dependent. In the unique environment of the ICU gut microbiota, this context-dependence is crucial and may lead to unexpected and even deleterious changes in gut microbiota composition. The observation that prior antibiotic exposure modifies the microbial response to scFOS-EN from beneficial to potentially harmful has important clinical implications. Together our results raise concern about the use of “one-size-fits-all” prebiotic strategies in the ICU, and call for context-aware, personalized nutritional interventions. Moving forward, identifying microbial signatures that predict responsiveness to microbiota-targeted therapies—and incorporating analytical approaches that capture temporal patterns and interspecies interactions—will be essential to develop safe and effective interventions in critical illness.

## Supplementary Information

Additional file 1: Completed CONSORT 2025 checklist.

Additional file 2: Full nutritional composition of Osmolite® 1.2 Cal and Vital® AF 1.2 Cal enteral formulas, including their macronutrient, micronutrient, and fiber content.

Additional file 3: Participant and group specific clinical data captured during hospitalization, including daily nutritional logs and antibiotics along with clinical outcomes. In addition, it provides the full list of clinical variables included in Spearman’s correlation analyses.

Additional file 4: Detailed fecal microbiota analysis data. LMMs of relative abundances at phylum-level and genera-level (separated into genera > 0.01% and rare genera (0.001–0.01%r.a.), raw relative abundance data at genera (all), TEMPTED outputs (combined and scFOS-EN specific data).

Additional file 5: Figures S1**–**S8. FigS1-Oral & Fecal Sample Distributions. FigS2-Alpha Diversity Measures in NF-EN vs scFOS-EN Fecal Samples. FigS3-LOESS plots for significant genera. FigS4-LMMs of Enterobacteriaceae family changes over time and Day 10 estimates. FigS5-Changes in Oral Microbial Dynamics in NF-EN vs scFOS-EN. FigS6-Characteristics of fecal samples used for NF-EN and scFOS EN Networks. FigS7-MicNet Dashboard NF-EN and scFOS-EN Network topography results. FigS8-NF-EN and scFOS-EN network analyses reveal shared and formula-specific features.

## Data Availability

Sequencing data generated in this study have been deposited in the NCBI Sequence Read Archive (SRA) under BioProject accession number PRJNA1358002 [43] (https://identifiers.org/ncbi/bioproject:PRJNA1358002). All other data supporting the findings of this study are available from the corresponding author upon reasonable request.

## Abbreviations

AE: Adverse event
AIC: Akaike Information Criterion
APACHE II: Acute Physiology and Chronic Health Evaluation II
ASV: Amplicon sequence variant
BMI: Body mass index
CI: Confidence interval
CLR: Center-log ratio
CONSORT: Consolidated Standards of Reporting Trials
DNA: Deoxyribonucleic acid
EN: Enteral nutrition
g: Grams
g/day: Grams per day
GI: Gastrointestinal
ICU: Intensive care unit
IMV: Invasive mechanical ventilation
IRB: Institutional review board
kcal: Kilocalories
kcal/day: Kilocalories per day
kcal/kg/day: Kilocalories per kilogram per day
kgAdj: Kilograms adjusted body weight
LMM: Linear mixed-effects model
LOESS: Locally estimated scatterplot smoothing
LOS: Length of stay
NF-EN: No-fiber enteral nutrition
PC: Principal component
PCR: Polymerase chain reaction
r.a.: Relative abundance
RCT: Randomized controlled trial
rRNA: Ribosomal ribonucleic acid
SCFA: Short-chain fatty acid
scFOS: Short-chain fructooligosaccharides
scFOS-EN: Short-chain fructooligosaccharides enteral nutrition
SparCC: Sparse correlations for compositional data
TEMPTED: TEMPoral TEnsor Decomposition
U: Unassigned (in the context of bacterial taxonomy, e.g., *U-Ruminococcaceae*)
