# Supplementary material for: Impact of fiber-containing enteral nutrition on microbial community dynamics in critically ill trauma patients: a pilot-randomized trial
Source: BMC Med. 2025 Dec 29;23:706. doi: 10.1186/s12916-025-04511-2 (PMC12751656; doi:10.1186/s12916-025-04511-2)
Supplement: Supplementary file 2 — Additional file 2: Full nutritional composition of Osmolite® 1.2 Cal and Vital® AF 1.2 Cal enteral formulas, including their macronutrient, micronutrient, and fiber content. [file 12916_2025_4511_MOESM2_ESM.pdf]

Detailed EN Formula Information

A comparison was conducted between Osmolite® 1.2 Cal and Vital® AF 1.2 Cal based on their nutritional content per 1-liter serving. Osmolite® 1.2 Cal provides 55 grams of protein, 38 grams of fat, and 156 grams of carbohydrates per serving. In contrast, Vital® AF 1.2 Cal contains 75 grams of protein, 54 grams of fat, and 111 grams of carbohydrates, and includes 1.2 grams of dietary fiber, sourced from NutraFlora® scFOS. NutraFlora® P-95 is a highly purified prebiotic fiber ingredient, containing a minimum of 95% short-chain fructooligosaccharides (scFOS), composed of DP3, DP4, and DP5 molecules. Per 100 grams, NutraFlora® P-95 provides 96.8 grams of carbohydrates, of which 92.5 grams is dietary fiber from scFOS. The remaining carbohydrates include 4.3 grams of sugars, with only 0.3 grams of fat and less than 0.1 grams of protein (detailed content of NutraFlora® P-95 is found below (see picture)).

Micronutrient Comparison Between Formulas:

Micronutrient	Osmolite® 1.2 Cal	Vital® AF 1.2 Cal
Vitamin A (mcg RAE)	171.0	365.0
Vitamin D (mcg)	3.8	6.1
Vitamin E (mg)	10.0	4.8
Vitamin K (mcg)	29.0	33.4
Vitamin C (mg)	59.0	43.0
Folate (mcg DFE)	76.0	124.0
Folic Acid (mcg)	46.0	74.0
Thiamin (mg)	0.41	0.6
Riboflavin (mg)	0.59	0.4
Vitamin B6 (mg)	0.33	0.55
Vitamin B12 (mcg)	2.3	1.65
Niacin (mg NE)	4.0	8.0
Choline (mg)	142.0	105.0
Biotin (mcg)	5.7	7.2
Pantothenic Acid (mg)	0.95	1.7
Sodium (mg)	253.0	300.0

Potassium (mg)	539.0	390.0
Chloride (mg)	356.0	300.0
Calcium (mg)	284.0	248.0
Phosphorus (mg)	284.0	238.0
Magnesium (mg)	88.0	80.0
Iodine (mcg)	36.0	30.0
Manganese (mg)	0.81	0.8
Copper (mg)	0.41	0.35
Zinc (mg)	2.5	4.8
Iron (mg)	3.4	3.6
Selenium (mcg)	14.0	15.0
Chromium (mcg)	14.0	16.0
Molybdenum (mcg)	23.0	25.6
Calories	285.0	284.0
L-Carnitine (mg)	36.0	30.0
Taurine (mg)	36.0	30.0
Water (mL)	195.0	192.0

technical specification

Ingredion

NUTRAFLORA® P-95 111001

NUTRAFLORA® P-95 prebiotic fiber contains a minimum of 95% (dry basis) of highly pure short-chain fructooligosaccharides (scFOS®) consisting of GF2, GF3 and GF4 molecules.

Chemical and Physical Properties

	Min.	Max.
Moisture, %	-	5.0
pH (10% solution)	5.0	7.0
Lead, ppm	-	1
Arsenic, ppm	-	0.2

Sensory Data

Appearance	White Powder
Odor	Standard, odorless
Flavor	Standard, clean slightly sweet

Typical Carbohydrate Profile, % d.b.

	Min.	Max.
Sugar (sucrose, glucose, & fructose)	-	5
Fructooligosaccharides (scFOS)	95	-
GF2 (DP3)	30	42
GF3 (DP4)	45	57
GF4 (DP5)	5	15

Screen Test

	Min.	Max.
% thru 40 mesh U.S.S.	100	-

Microbiological Limits

	Max.
Standard Plate Count, cfu/g	300
Yeast, cfu/g	20
Mold, cfu/g	20
Coliforms, cfu/g	10
Salmonella	Negative
E.coli	Negative
Staphylococcus aureus	Negative
Anaerobic Thermophilic Spores, cfu/g	10
Aerobic Thermophilic Spores, cfu/g	10
Anaerobic Mesophilic Spores, cfu/g	10
Aerobic Mesophilic Spores, cfu/g	10
Listeria	Negative
Cronobacter Sakazakii	Negative

Nutritional Data/100 g

	Typical
Calories	200
Calories from fat	2
Total Fat, g	0.3
Cholesterol, mg	0
Sodium, mg	2
Total Carbohydrate, g	96.8
Dietary Fiber**, g	92.5
Total Sugars***, g	4.3
Added Sugars, g	0
Other Carbohydrate, g	0
Protein, g	<0.1*
Vitamin D, mcg	0
Calcium mg	<2*
Iron, mg	<0.2*
Potassium, mg	<10*
Ash, g	<0.1*

Certification

Kosher pareve, Halal

Packaging and Storage

Bags
Supersacs

Recommended handling and storage temperature is 25˚C (77˚F) and 33% relative humidity.

Shelf Life

The best before date for NUTRAFLORA® P-95 is 24 months from the date of manufacture when stored under proper conditions.

Regulatory Data

CAS No. 308066-66-2

United States

GRAS GRN No. 1006
FCC (Food Chemical Codex) monograph for fructooligosaccharides

Labeling: FOS, fructooligiosaccharides, short-chain fructooligosaccharides, scFOS, short chain fructan, short chain fructan fiber, cane sugar/sugar derived short chain fructan fiber,

Canada

Health Canada approved as ingredient and fiber
Labeling: Fructooligosaccharides, oligofructose

Features and Benefits

NUTRAFLORA® P-95 is a prebiotic fiber that promotes digestive, immune & bone health. It selectively stimulates the growth and/or activity of beneficial microflora while being unavailable to pathogens. It has demonstrated effects in published peer-reviewed human studies. It contributes to dietary fiber, has a low caloric contribution, direct compressible and is heat stable. It has a clean slightly sweet flavor profile and performs similarly to sugar at typical inclusion levels. The ingredient is soluble and does not contribute to viscosity. It enhances flavors- rounds & extends the sweetness profile of high intensity sweeteners.

* Not present at level of quantification.
** Dietary Fiber=fructooligosaccharides (scFOS) with a DP of 3-5
*** "Total Sugars" in this product may contribute to "Added Sugars" for nutrition labeling purposes in the final consumer product.

Effective Date: February 25, 2025   Next Review Date: February 25, 2028

The information described above is offered solely for your consideration, investigation, and independent verification. It is up to you to decide whether and how to use this information. Ingredion Incorporated and the Ingredion group of companies make no warranty about the accuracy or completeness of the information contained above or the suitability of any of their products for your specific intended use. Furthermore, all express or implied warranties of noninfringement, merchantability, or fitness for a particular purpose are hereby disclaimed. Ingredion Incorporated and the Ingredion group of companies assume no responsibility for any liability or damages arising out of or relating to any of the foregoing.

5 Westbrook Corporate Ctr.
Westchester, Illinois 60154
U.S.A.
Ph: 708.551.2600

1600-90 Burnhamthorpe Road West,
Mississauga, Ontario L5B 0H9
Canada
Ph: 905.281.7950

www.ingredion.us

The INGREDION mark and logo are trademarks of the Ingredion group of companies. All rights reserved.
All contents copyright © 2025.
