# Supplementary material for: Impact of fiber-containing enteral nutrition on microbial community dynamics in critically ill trauma patients: a pilot-randomized trial
Source: BMC Med. 2025 Dec 29;23:706. doi: 10.1186/s12916-025-04511-2 (PMC12751656; doi:10.1186/s12916-025-04511-2)
Supplement: Supplementary file 5 — Additional file 5: Figures S1–S8. FigS1-Oral & Fecal Sample Distributions. FigS2-Alpha Diversity Measures in NF-EN vs scFOS-EN Fecal Samples. FigS3-LOESS plots for significant genera. FigS4-LMMs of Enterobacteriaceae family changes over time and Day 10 estimates. FigS5-Changes in Oral Microbial Dynamics in NF-EN vs scFOS-EN. FigS6-Characteristics of fecal samples used for NF-EN and scFOS EN Networks. FigS7-MicNet Dashboard NF-EN and scFOS-EN Network topography results. FigS8-NF-EN and scFOS-EN network analyses reveal shared and formula-specific features. [file 12916_2025_4511_MOESM5_ESM.pdf]

SI File S4 - Supplemental Figures

SF1: Oral & Fecal Sample Distributions

SF2: Alpha Diversity Measures in NF-EN vs scFOS-EN Fecal Samples

SF3: LOESS plots for significant genera

SF4: LMMs of *Enterobacteriaceae* family changes over time and Day 10 estimates

SF5: Changes in Oral Microbial Dynamics in NF-EN vs scFOS-EN

SF6: Characteristics of fecal samples used for NF-EN and scFOS-EN Networks

SF7: MicNet Dashboard NF-EN and scFOS-EN Network topography results

SF8: NF-EN and scFOS-EN network analyses reveal shared and formula-specific features

Supplemental Figure 1

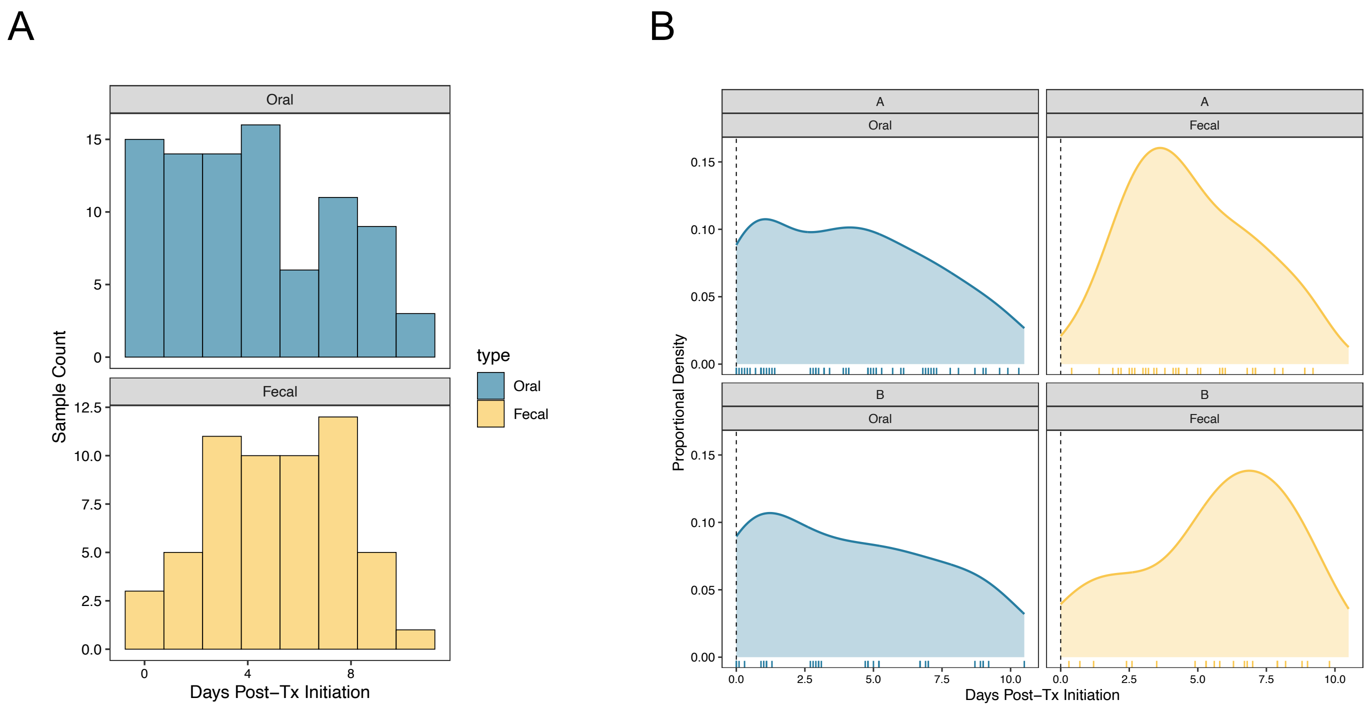

SF1: Oral & Fecal Sample Distributions

A) Distribution of numbers of oral (top) and fecal (bottom) samples by collection time for samples included in final analysis . B) Proportional density of oral (left) and fecal (right) samples by collection time for scFOS-EN (Group A, top) and NF-EN (Group B, bottom)

Supplemental Figure 2

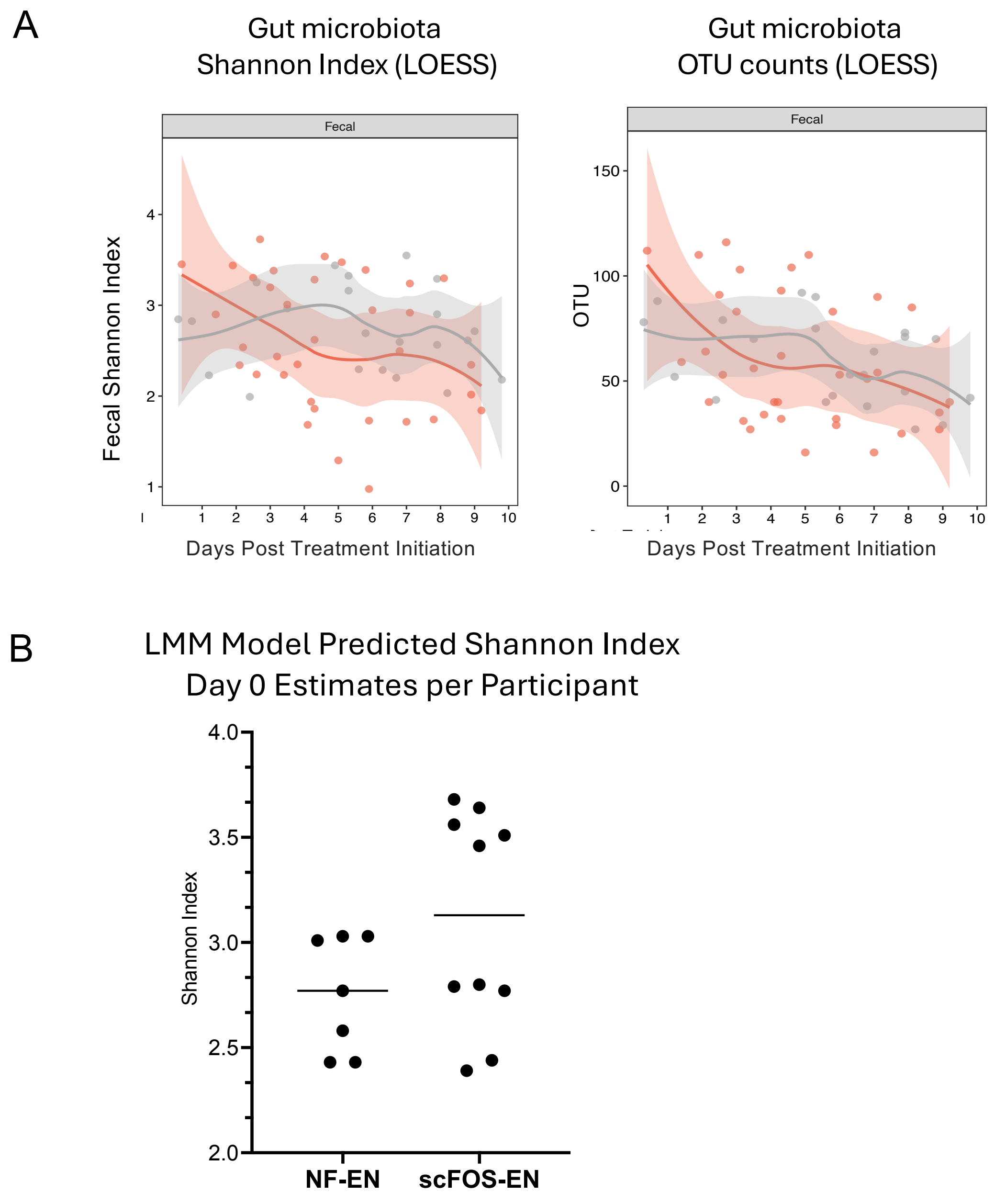

SF2: Alpha Diversity Measures in NF-EN vs scFOS-EN Fecal Samples

A) Locally-estimated scatterplot smoothing (LOESS) of OTUs (α-diversity) from NF-EN and scFOS-EN fecal samples over study period. B) "D0 Shannon estimates" based on LMM modeling for each participant, grouped by NF-EN and scFOS-EN.

Supplemental Figure 3

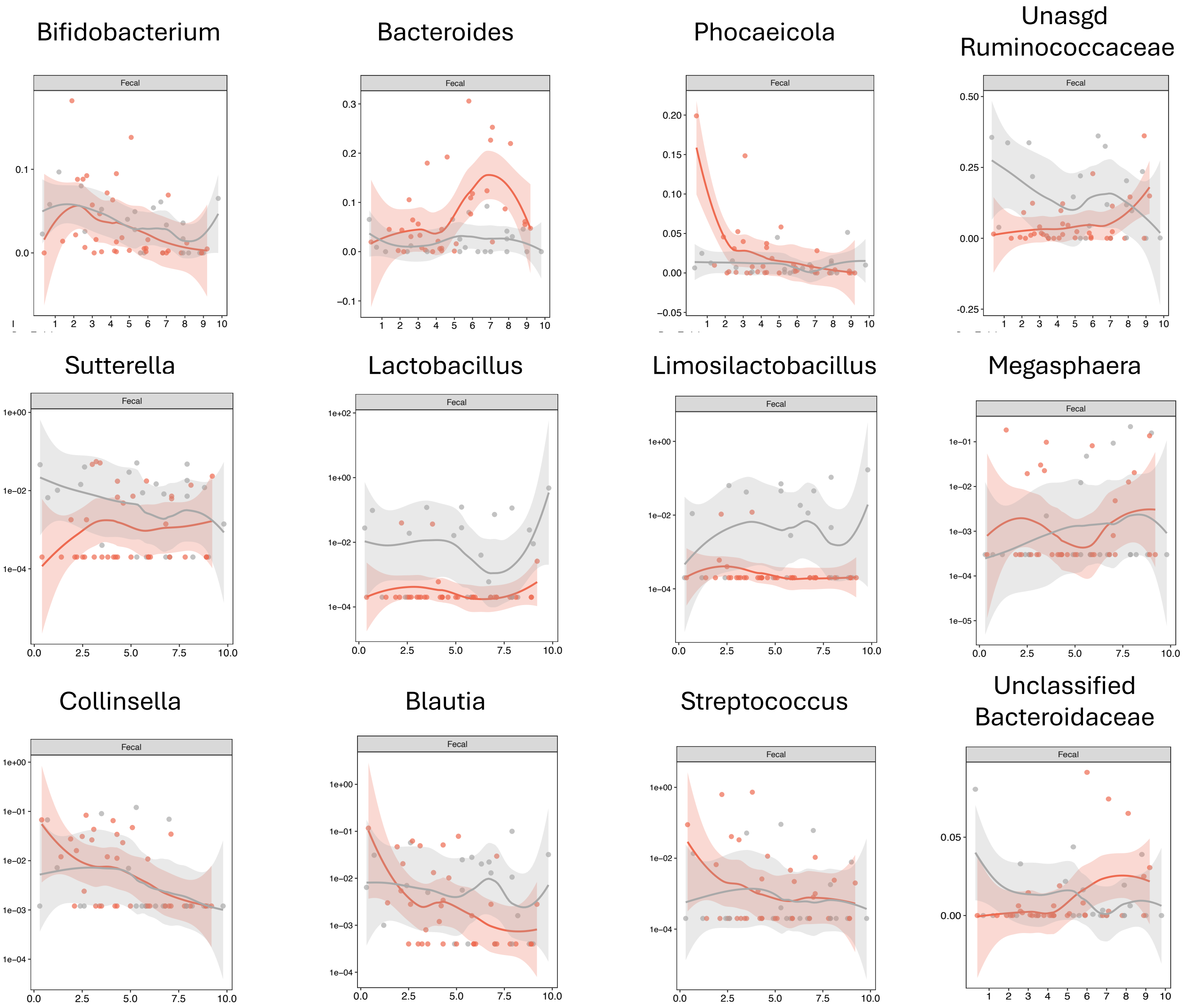

SF3: NF-EN vs scFOS-EN Fecal – LOESS plots for significant genera

LOESS visualization of commensal microbes identified to significantly differ over study period (see main Figure 3 and SI File S3 for significance and full outputs of linear mixed models).

Supplemental Figure 4

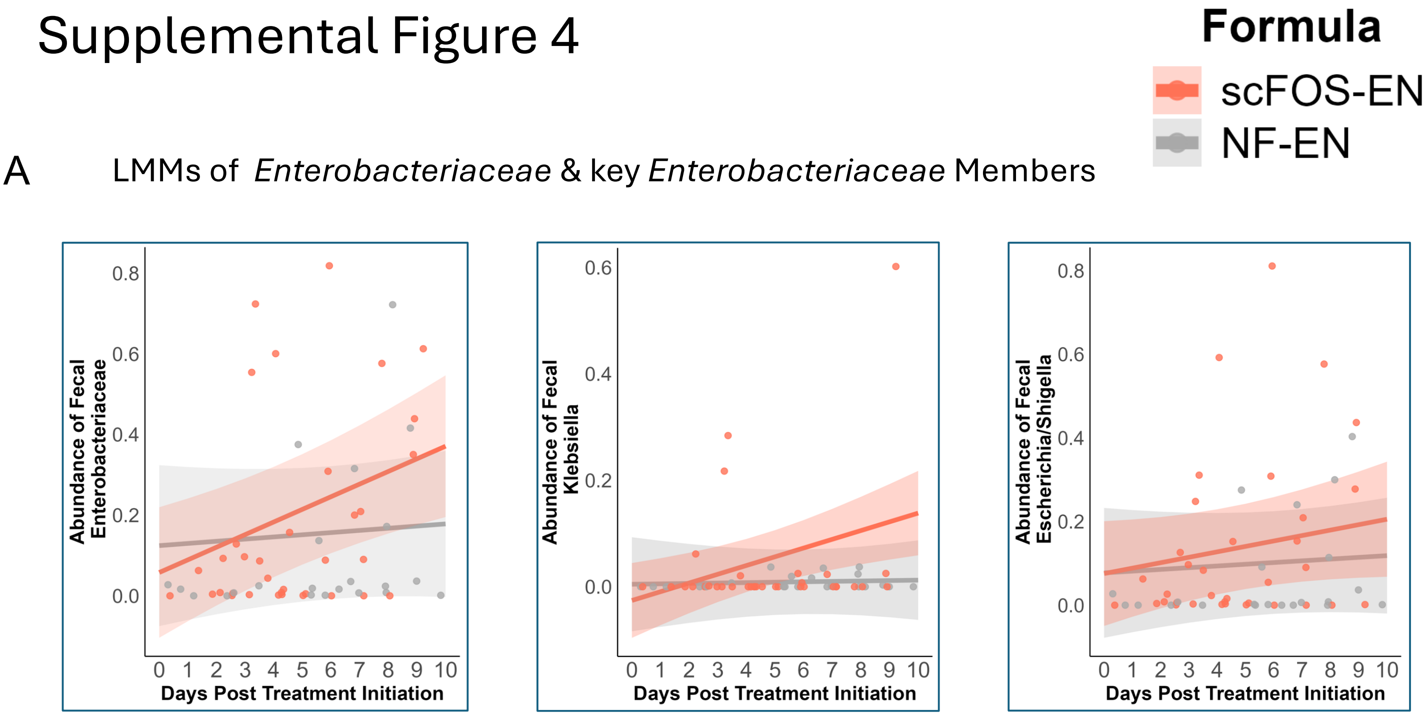

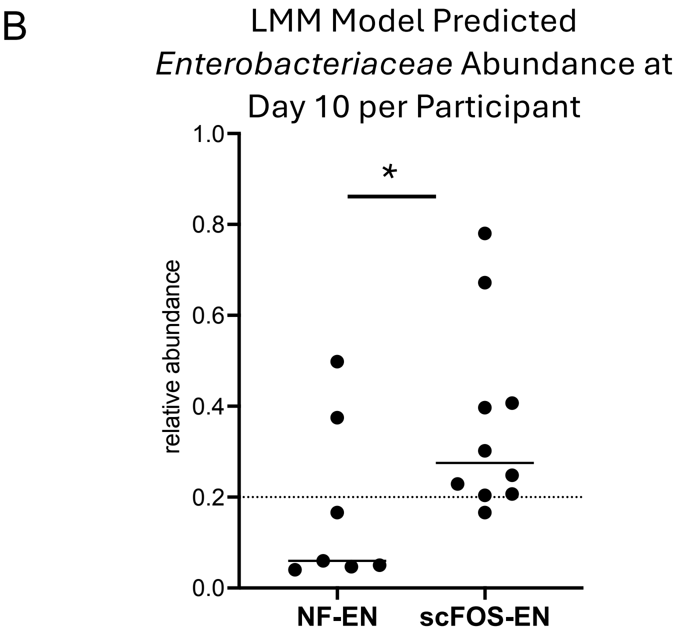

SF4: LMMs of *Enterobacteriaceae* family changes over time and Day 10 estimates

A) Top left to right: LMMs of *Enterobacteriaceae* (family), *Klebsiella* (genus), and *Escherichia-Shigella* (genus). B) LMM predicted estimates of *Enterobacteriaceae* family at Day 10 based on participant-level extrapolations.

Supplemental Figure 5

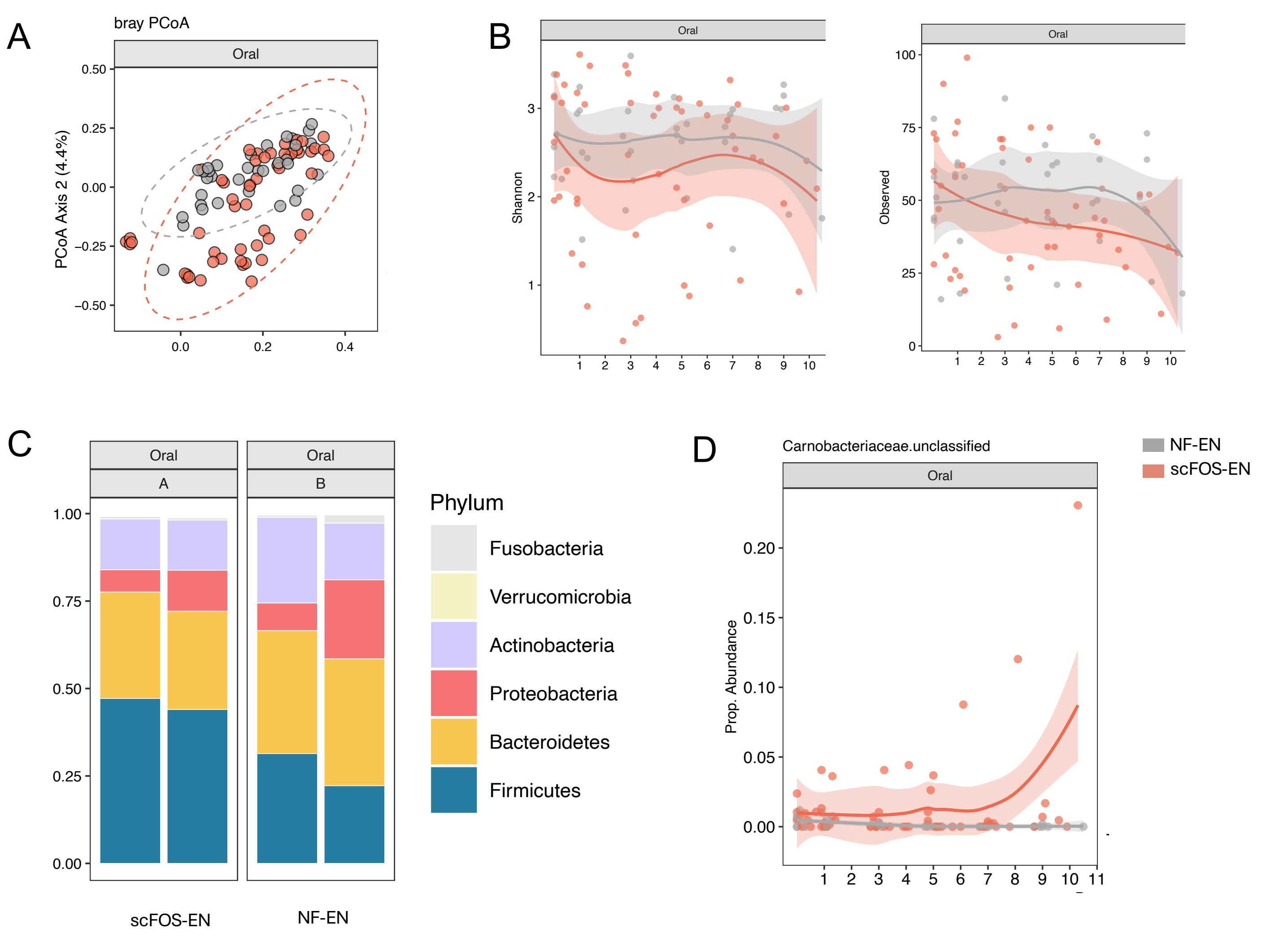

	scFOS-EN+		NF-EN		Difference (scFOS-NF) Between Groups	
	Estimate (95% CI)	p-value	Estimate (95% CI)	p-value	Estimate (95% CI)	p-value
Carnobacteriaceae.unclassified	0.003 (0.001, 0.005)	0.0045	-0.001 (-0.003, 0.002)	0.6208	0.003 (0, 0.006)	0.0273
Fusobacterium	0 (-0.002, 0.002)	0.8237	0.003 (0, 0.005)	0.0190	-0.003 (-0.005, 0)	0.0552
Prevotellaceae.unclassified	0 (-0.004, 0.004)	0.8981	0.002 (-0.002, 0.007)	0.3067	-0.003 (-0.008, 0.003)	0.3953
Schaalia	0 (-0.003, 0.004)	0.8978	-0.004 (-0.008, -0.001)	0.0284	0.005 (-0.001, 0.01)	0.0826
Veillonella	-0.008 (-0.019, 0.003)	0.1493	-0.014 (-0.026, -0.002)	0.0238	0.006 (-0.01, 0.022)	0.4422

SF5: Changes in Oral Microbial Dynamics in NF-EN vs scFOS-EN

A) PcOA of Bray-Curtis distance measures B) LOESs of Shannon and OTU measures over study period (alpha-diversity) C) Taxonomic composition at phylum-level with day 0-2 represented in first columns of each group, days 2-10 in 2nd column. D) Results of LMMRM analysis at genus-level. Estimates demonstrated as change in r.a. per day (Δ r.a./day) for changes over time within each group and differences in rate of change over time across groups (last 2 columns). Data from all NF-EN & scFOS-EN samples (n=35 & n=53 respectively).

Supplemental Figure 6

A

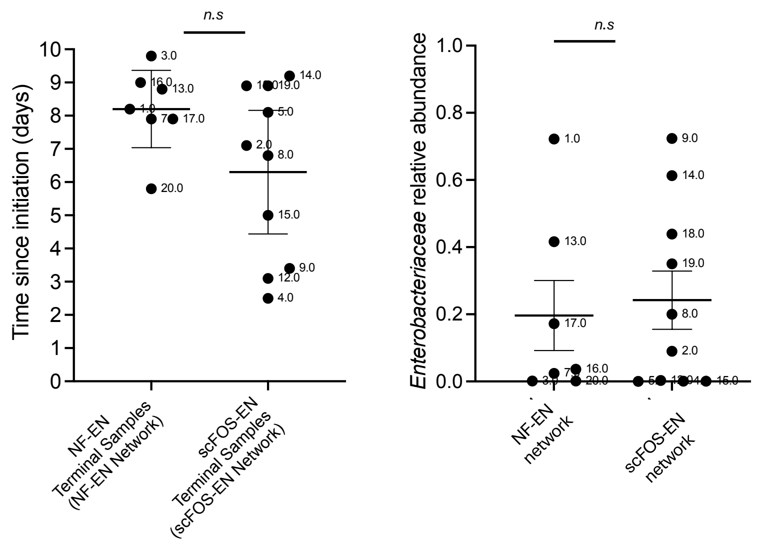

B

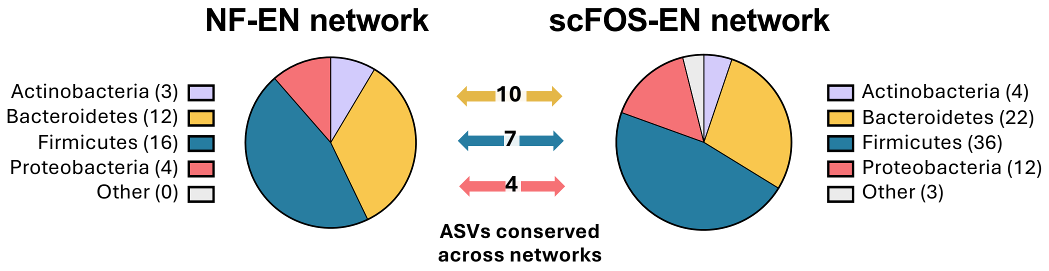

SF6: Characteristics of fecal samples used for NF-EN and scFOS-EN Networks

A) Comparison of sample collection timing (left) and *Enterobacteriaceae* relative abundance (right) between NF-EN and scFOS-EN fecal samples used in construction of networks. MW comparisons shows no significant difference in comparisons; data expressed as mean +/- SEM, points labeled by patient ID. B) Distribution of phylum-level taxonomic composition of all ASVs (nodes) in each network and number of ASVs from each phyla present in both networks (3 of 4 Proteobacteria ASVs belong to *Enterobacteriaceae* family).

Supplemental Figure 7

NF-EN Network Topography

Large-scale metrics of network

	Basic network information
Nodes	35.0000
Total interactions	78.0000
Positive interactions	45.0000
Negative interactions	33.0000
Pos-Neg ratio	1.3636
Density	0.1294
Average degree	4.4000
Degree std	1.6423
Components	2.0000
Diameter	7.0000
Clustering coefficient	0.3724
Shortest average path length	2.7538
Modularity	0.5752
Small-world index	2.8864

Basic structural balance information

	Structural balance
Percentage balanced	1.0000
Percentage unbalanced	0.0000
Triangles +++	0.4800
Triangles --+	0.5200
Triangles ++-	0.0000
Triangles ---	0.0000

scFOS-EN Network Topography

Large-scale metrics of network

	Basic network information
Nodes	80.0000
Total interactions	337.0000
Positive interactions	160.0000
Negative interactions	177.0000
Pos-Neg ratio	0.9040
Density	0.1063
Average degree	8.4000
Degree std	4.2267
Components	2.0000
Diameter	5.0000
Clustering coefficient	0.3319
Shortest average path length	2.4200
Modularity	0.4390
Small-world index	2.9011

Basic structural balance information

	Structural balance
Percentage balanced	1.0000
Percentage unbalanced	0.0000
Triangles +++	0.2411
Triangles --+	0.7589
Triangles ++-	0.0000
Triangles ---	0.0000

SF7: MicNet Dashboard NF-EN and scFOS-EN Network topography results

Results from MicNet Dashboard of NF-EN (left) and scFOS-EN (right) network analyses; outputs demonstrate global network features including information on network topology (top) and structural information on the connections between nodes (bottom).

Supplemental Figure 8

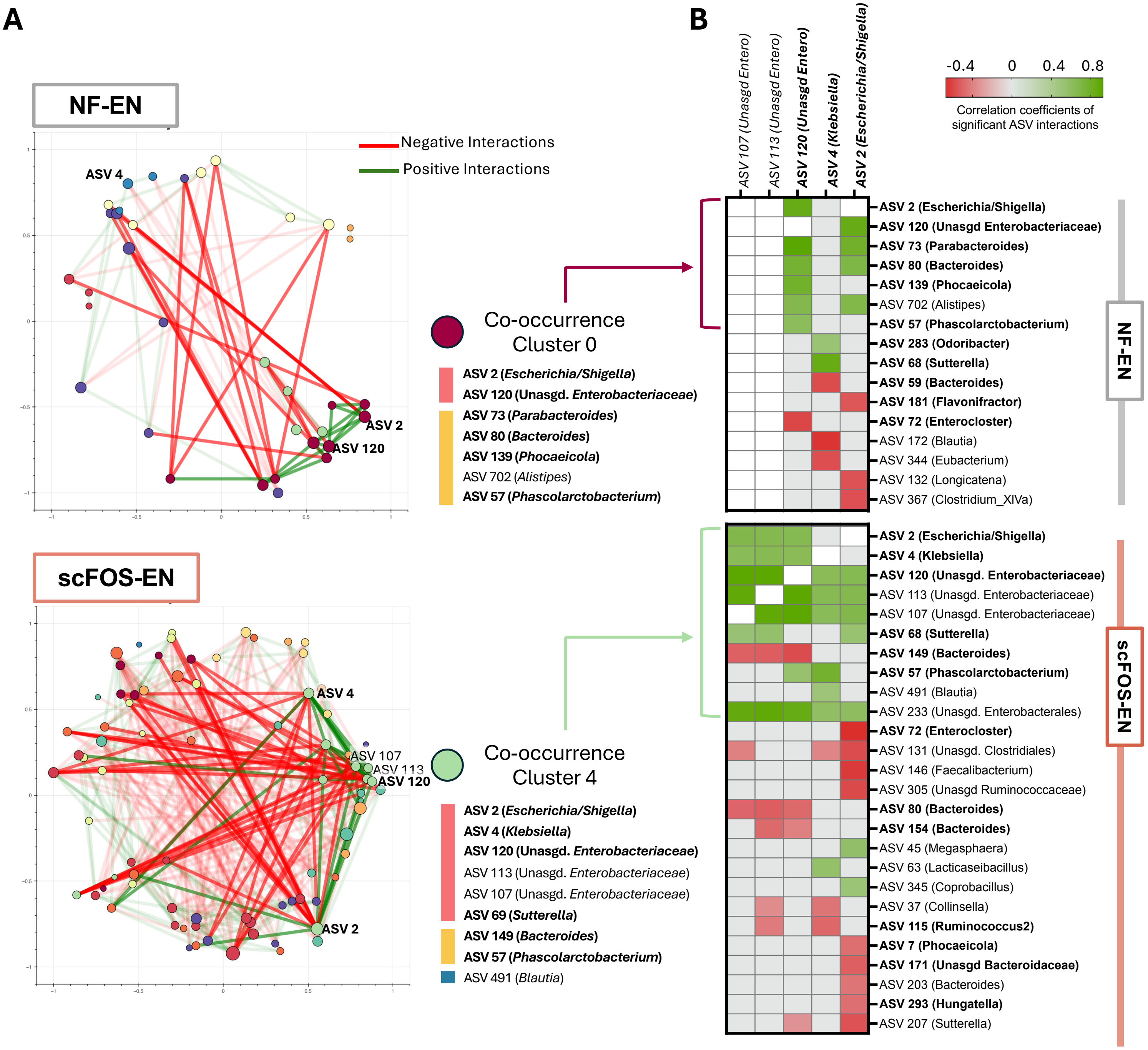

SF8: NF-EN and scFOS-EN network analyses reveal shared and formula-specific features

A) Network analyses generated using MicNet Toolbox of NF-EN and scFOS-EN networks. In both network figures, ASVs that map to *Enterobacteriaceae* are in bold and their connections to other ASVs (nodes) in the network are highlighted; note greater representation of negative correlations (red) in scFOS-EN. Nodes are colored by the co-occurrence cluster assigned by unsupervised hierarchical clustering (Louvain), with members of the *Enterobacteriaceae*- cluster identified in both NF-EN (Cluster 0) and scFOS-EN networks (Cluster 4) denoted to right, using genus-level assignments and phylum-level coloring **B)** Heat maps of *Enterobacteriaceae* interactions in NF-EN network (top) and scFOS-EN network (bottom), demonstrating only significant (q<0.1) SparCC correlations between *Enterobacteriaceae* ASVs (columns) and other ASVs in the network (rows); ASVs in bold present in both networks.
